# Supplementary material for: 1H NMR Chemical Exchange Techniques Reveal Local and Global Effects of Oxidized Cytosine Derivatives
Source: ACS Phys Chem Au. 2022 Feb 11;2(3):237–46. doi: 10.1021/acsphyschemau.1c00050 (PMC9137243; doi:10.1021/acsphyschemau.1c00050)
Supplement: Supplementary file 1 — pg1c00050_si_001.pdf [file pg1c00050_si_001.pdf]

Supporting Information: ^{1}H NMR chemical exchange techniques reveal local and global effects of oxidized cytosine derivatives

Romeo C. A. Dubini,[†,‡] Eva Korytiaková,[†] Thea Schinkel,[†,§] Pia Heinrichs,[†,§] Thomas Carell,[†] and Petra Rovó[*,†,‡,¶]

†*Faculty of Chemistry and Pharmacy, Department of Chemistry, Ludwig-Maximilians-Universität München, Butenandstraße 5-13, 81377 Munich, Germany*

‡*Center for Nanoscience (CeNS), Faculty of Physics, Ludwig-Maximilians-Universität München, Schellingstraße 4, 5th floor, 80799 Munich, Germany*

¶*Institute of Science and Technology (IST), Am Campus 1, 3400 Klosterneuburg, Austria*

§*Contributed equally to this work*

E-mail: petra.rovo@ist.ac.at

List of Figures

S1 ^{1}H-^{13}C HSQC aromatic region spectrum of f$C_{7.0}$, ca$C_{7.0}$ and $C_{7.0}$ S3

S2 ^{1}H-^{15}N HMQC imino region spectrum of f$C_{7.0}$, ca$C_{7.0}$ and $C_{7.0}$ S4

S3 ^{1}H-^{15}N HMQC imino region spectrum of ca$C_{7.0}$, ca$C_{5.8}$ and ca$C_{4.7}$ S5

S4 ^{1}H-^{13}C HSQC aromatic region spectrum of ca$C_{7.0}$, ca$C_{5.8}$ and ca$C_{4.7}$ S6

S5 ^{1}H-^{1}H NOESY imino region spectrum of ca$C_{7.0}$, ca$C_{5.8}$ and ca$C_{4.7}$ S7

S6 ^{1}H-^{1}H NOESY aromatic region spectrum of ca$C_{7.0}$, ca$C_{5.8}$ and ca$C_{4.7}$ S8

S7 Comparison of ^{1}H chemical shift perturbations for all samples, 1/2 . . . . . . S9

S8 Comparison of ^{1}H chemical shift perturbations for all samples, 2/2 . . . . . . S10

S9 Comparison of aromatic ^{13}C chemical shift perturbations for all samples . . . S11

S10 Comparison of aromatic ^{15}N chemical shift perturbations for all samples . . . S11

S11 Temperature-dependent CEST profiles and van't Hoff plots – C2H6 . . . . . S12

S12 Temperature-dependent CEST profiles and van't Hoff plots – A4H2 . . . . . S13

S13 Temperature-dependent CEST profiles and van't Hoff plots – A4H8 . . . . . S14

S14 Temperature-dependent CEST profiles and van't Hoff plots – T5H6 . . . . . S15

S15 Temperature-dependent CEST profiles and van't Hoff plots – A8H2 . . . . . S16

S16 Temperature-dependent CEST profiles and van't Hoff plots – A8H8 . . . . . S17

S17 Temperature-dependent CEST profiles and van't Hoff plots – T9H6 . . . . . S18

S18 Temperature-dependent CEST profiles and van't Hoff plots – C12H6 . . . . S19

S19 Concentration-dependent UV/Vis melting profiles S19

S20 On-resonance $R_{1\rho}$ relaxation dispersion profiles – $C_{7.0}$ S20

S21 On-resonance $R_{1\rho}$ relaxation dispersion profiles – ca$C_{7.0}$ S21

S22 On-resonance $R_{1\rho}$ relaxation dispersion profiles – ca$C_{5.8}$ S22

S23 On-resonance $R_{1\rho}$ relaxation dispersion profiles – ca$C_{4.7}$ S23

S24 On-resonance $R_{1\rho}$ relaxation dispersion profiles – f$C_{7.0}$ S24

List of Tables

S1 $caC_{7.0}$ CEST fitted relaxation and chemical shift parameters S25

S2 $caC_{5.8}$ CEST fitted relaxation and chemical shift parameters S27

S3 $caC_{4.7}$ CEST fitted relaxation and chemical shift parameters S29

S4 $caC_{7.0}$ dsDNA populations, kinetic rates and equilibrium constants S31

S5 $caC_{5.8}$ dsDNA populations, kinetic rates and equilibrium constants S33

S6 $caC_{4.7}$ dsDNA populations, kinetic rates and equilibrium constants S35

S7 CEST-derived thermodynamic and kinetic parameters S37

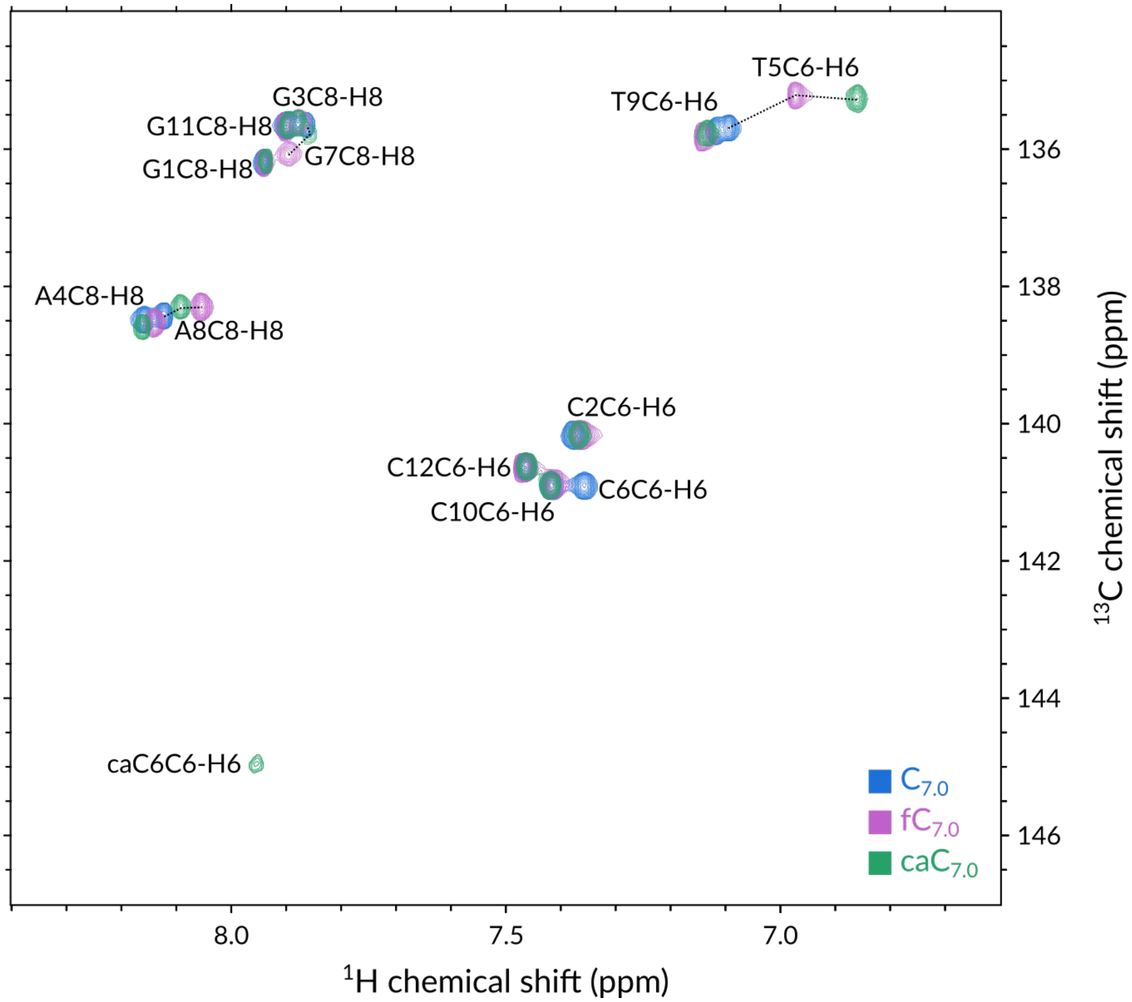

Figure S1: ^{1}H-^{13}C HSQC aromatic region spectrum of $fC_{7.0}$, $caC_{7.0}$ and $C_{7.0}$ recorded at 800 MHz at 37 ℃.

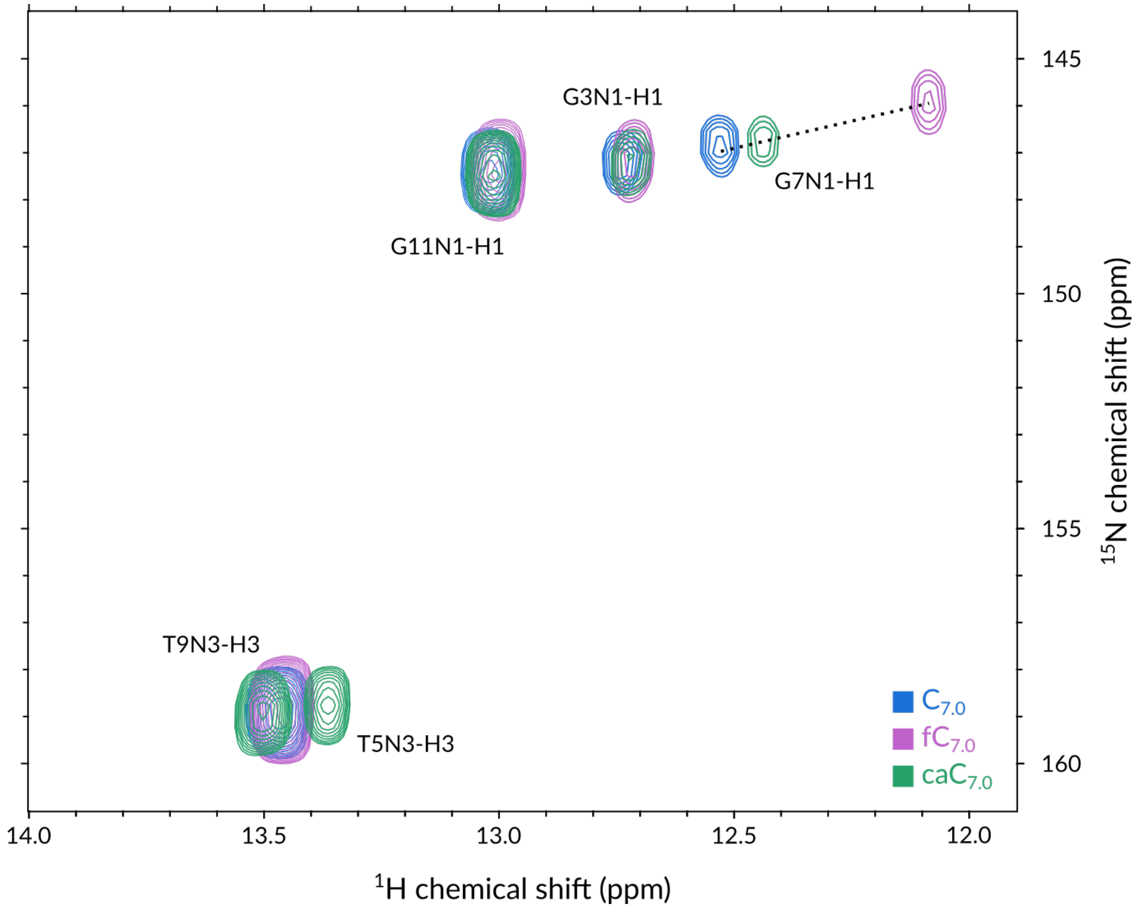

Figure S2: ^{1}H-^{15}N HMQC imino region spectrum of fC$_{7.0}$, caC$_{7.0}$ and C$_{7.0}$ recorded at 800 MHz at 37 °C.

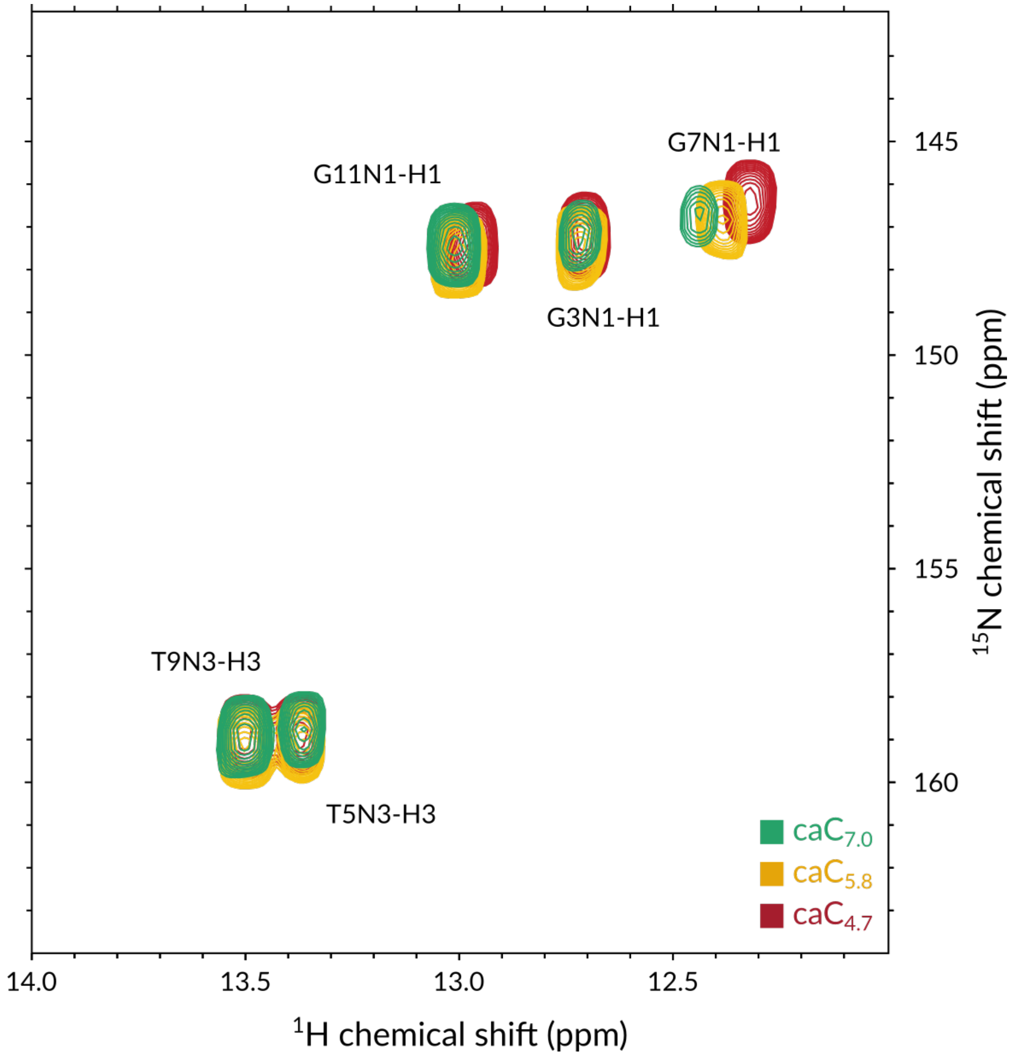

Figure S3: ^{1}H-^{15}N HMQC imino region spectrum of caC$_{7.0}$, caC$_{5.8}$ and caC$_{4.7}$ recorded at 800 MHz at 37 ℃.

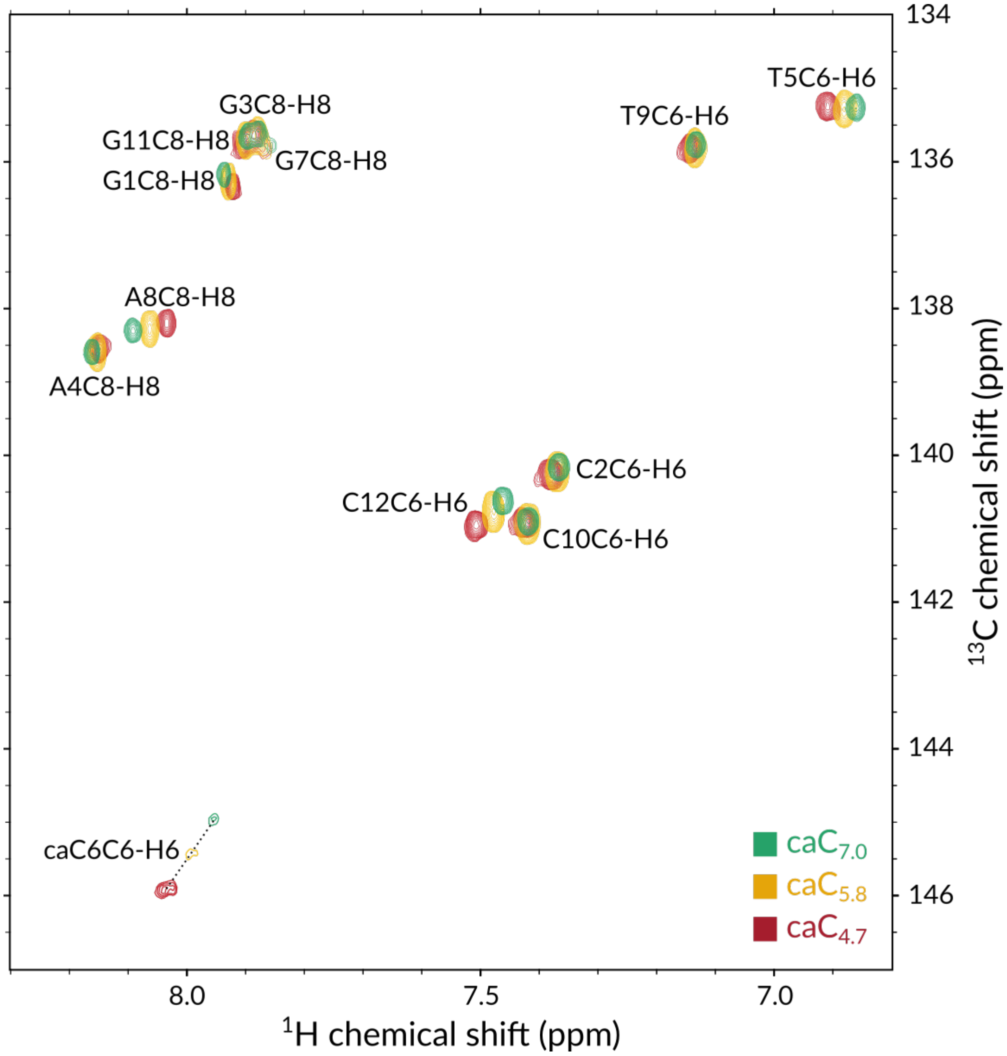

Figure S4: ^{1}H-^{13}C HSQC aromatic region spectrum of caC$_{7.0}$, caC$_{5.8}$ and caC$_{4.7}$ recorded at 800 MHz at 37 °C.

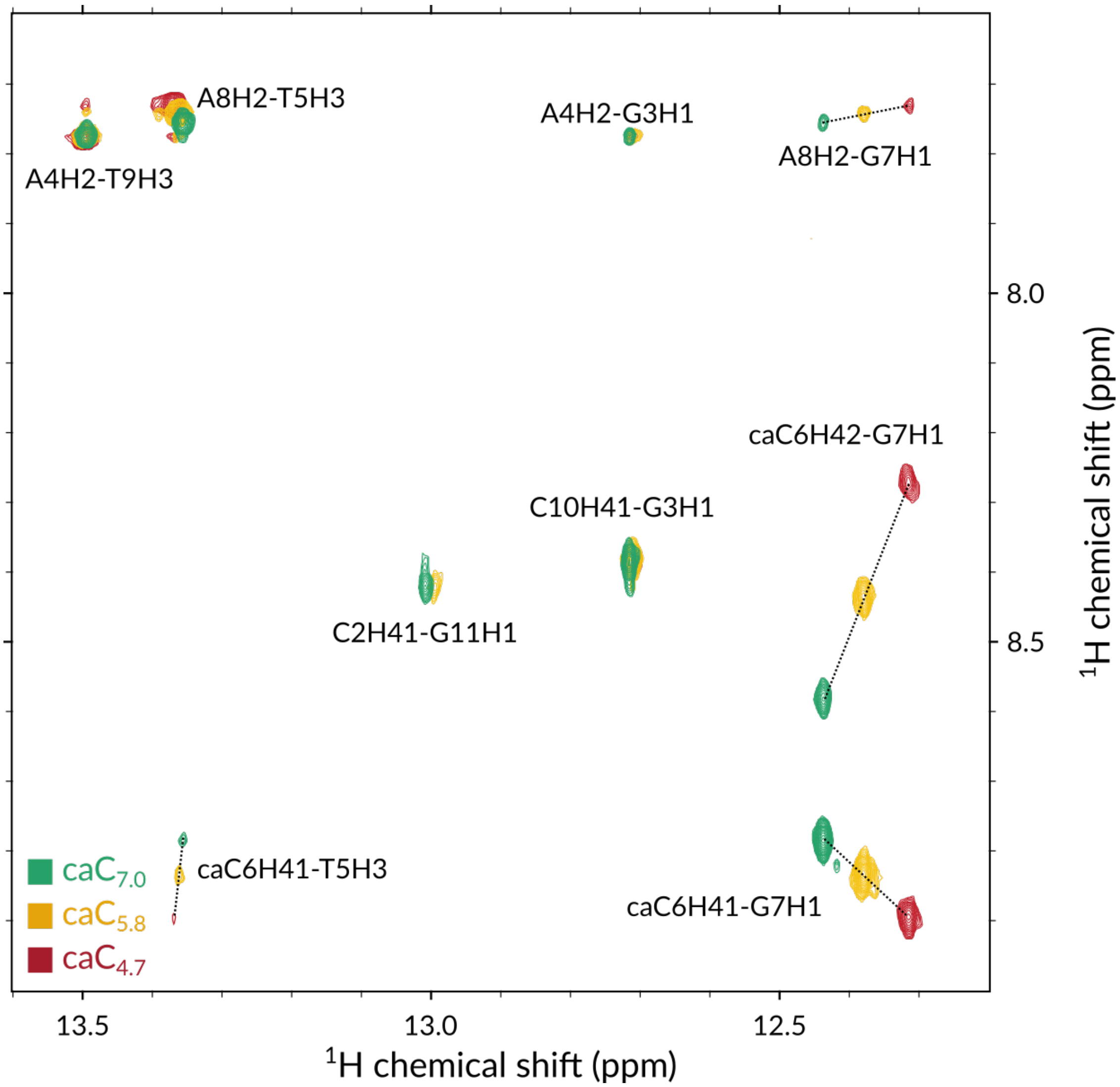

Figure S5: ^{1}H-^{1}H NOESY imino region spectrum of caC$_{7.0}$, caC$_{5.8}$ and caC$_{4.7}$ recorded at 800 MHz at 37 ℃.

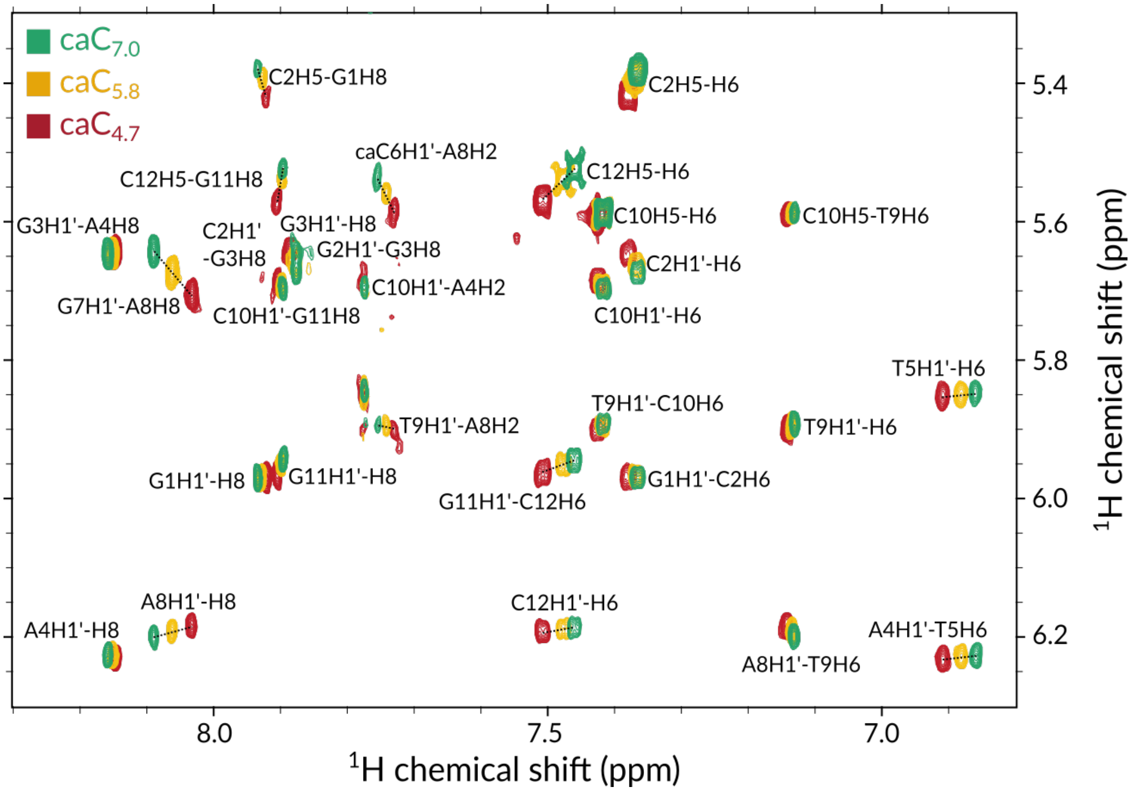

Figure S6: ^{1}H-^{1}H NOESY aromatic region spectrum of caC$_{7.0}$, caC$_{5.8}$ and caC$_{4.7}$ recorded at 800 MHz at 37 °C.

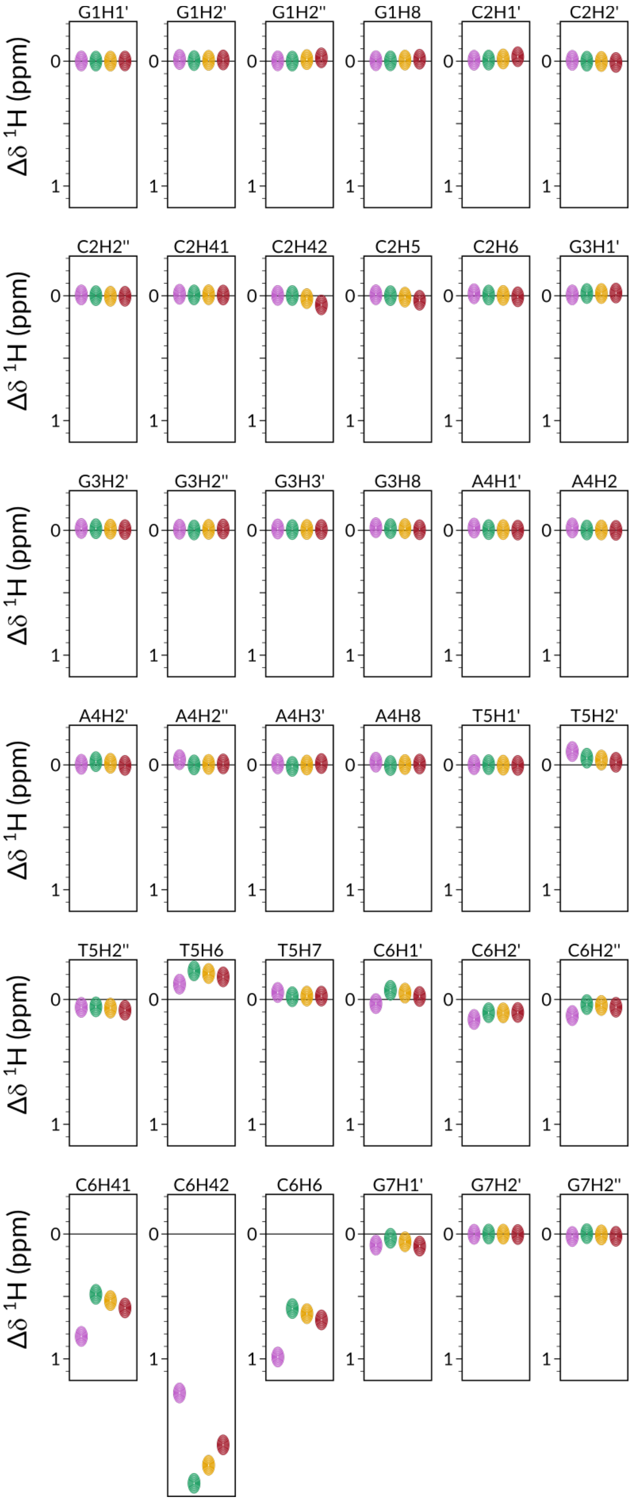

Figure S7: Comparison of chemical shift perturbations $\Delta\delta$ for all comparable aliphatic and aromatic ^{1}H nuclei across all samples in identical conditions. Resonances are reported with respect to the chemical shifts of $C_{7.0}$. Magenta, green, yellow and red symbols represent $fC_{7.0}$, $caC_{7.0}$, $caC_{5.8}$ and $caC_{4.7}$, respectively. Remainder of the figure follows in Fig. S3.

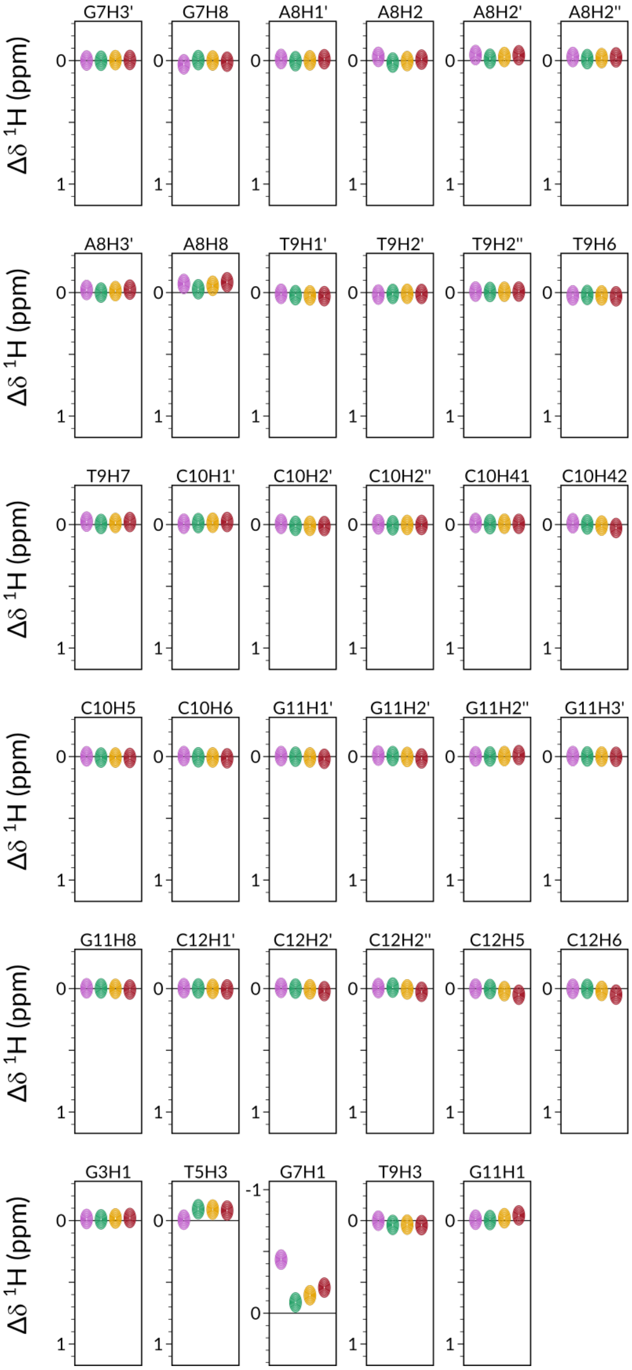

Figure S8: Comparison of chemical shift perturbations $\Delta\delta$ for all comparable aliphatic and aromatic ^{1}H nuclei across all samples in identical conditions. Resonances are reported with respect to the chemical shifts of $C_{7.0}$. Magenta, green, yellow and red symbols represent $fC_{7.0}$, $caC_{7.0}$, $caC_{5.8}$ and $caC_{4.7}$, respectively.

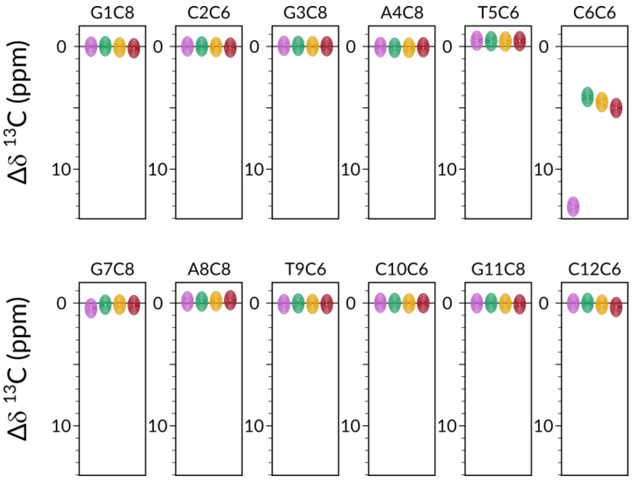

Figure S9: Comparison of chemical shift perturbations $\Delta\delta$ for all comparable aromatic ^{13}C nuclei across all samples in identical conditions. Resonances are reported with respect to the chemical shifts of $C_{7.0}$. Magenta, green, yellow and red symbols represent $fC_{7.0}$, $caC_{7.0}$, $caC_{5.8}$ and $caC_{4.7}$, respectively.

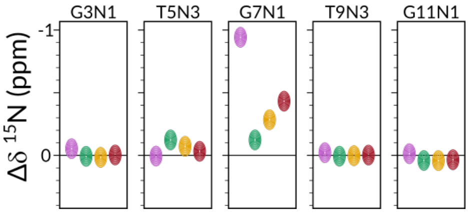

Figure S10: Comparison of chemical shift perturbations $\Delta\delta$ for all comparable aromatic ^{15}N nuclei across all samples in identical conditions. Resonances are reported with respect to the chemical shifts of $C_{7.0}$. Magenta, green, yellow and red symbols represent $fC_{7.0}$, $caC_{7.0}$, $caC_{5.8}$ and $caC_{4.7}$, respectively.

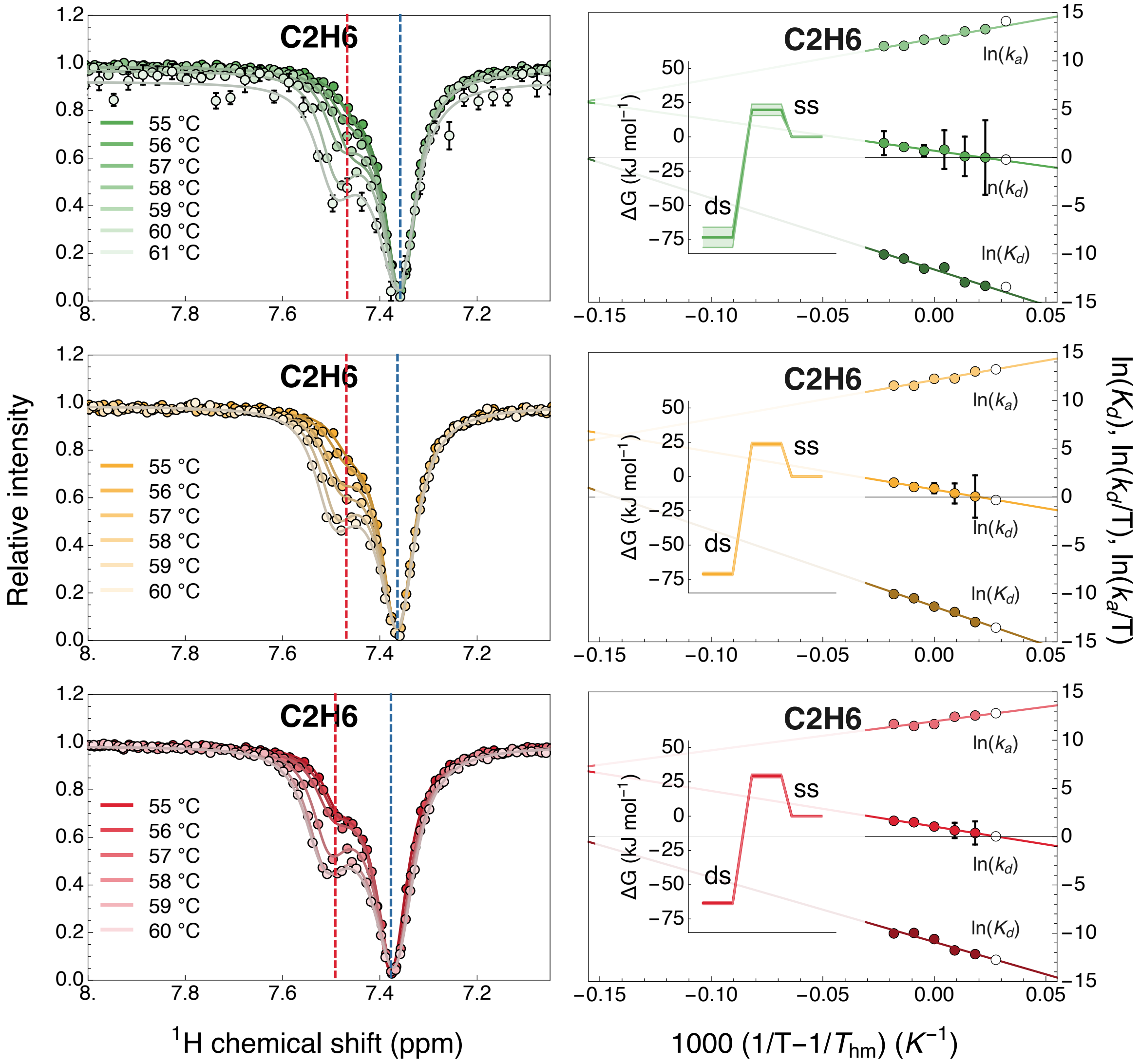

Figure S11: Temperature-dependent CEST melting profiles and van't Hoff plots for proton C2H6. caC$_{7.0}$, caC$_{5.8}$ and caC$_{4.7}$ are shown in shades of green, yellow and red, respectively. Dashed blue and red lines indicate GS and ES chemical shift values. In the van't Hoff plots, shades of green, yellow and red indicate data entries and linear fits for caC$_{7.0}$, caC$_{5.8}$ and caC$_{4.7}$, respectively. White data points represent back-calculated values. Insets present the relevant Gibbs free energy plots at each pH condition at 37 °C.

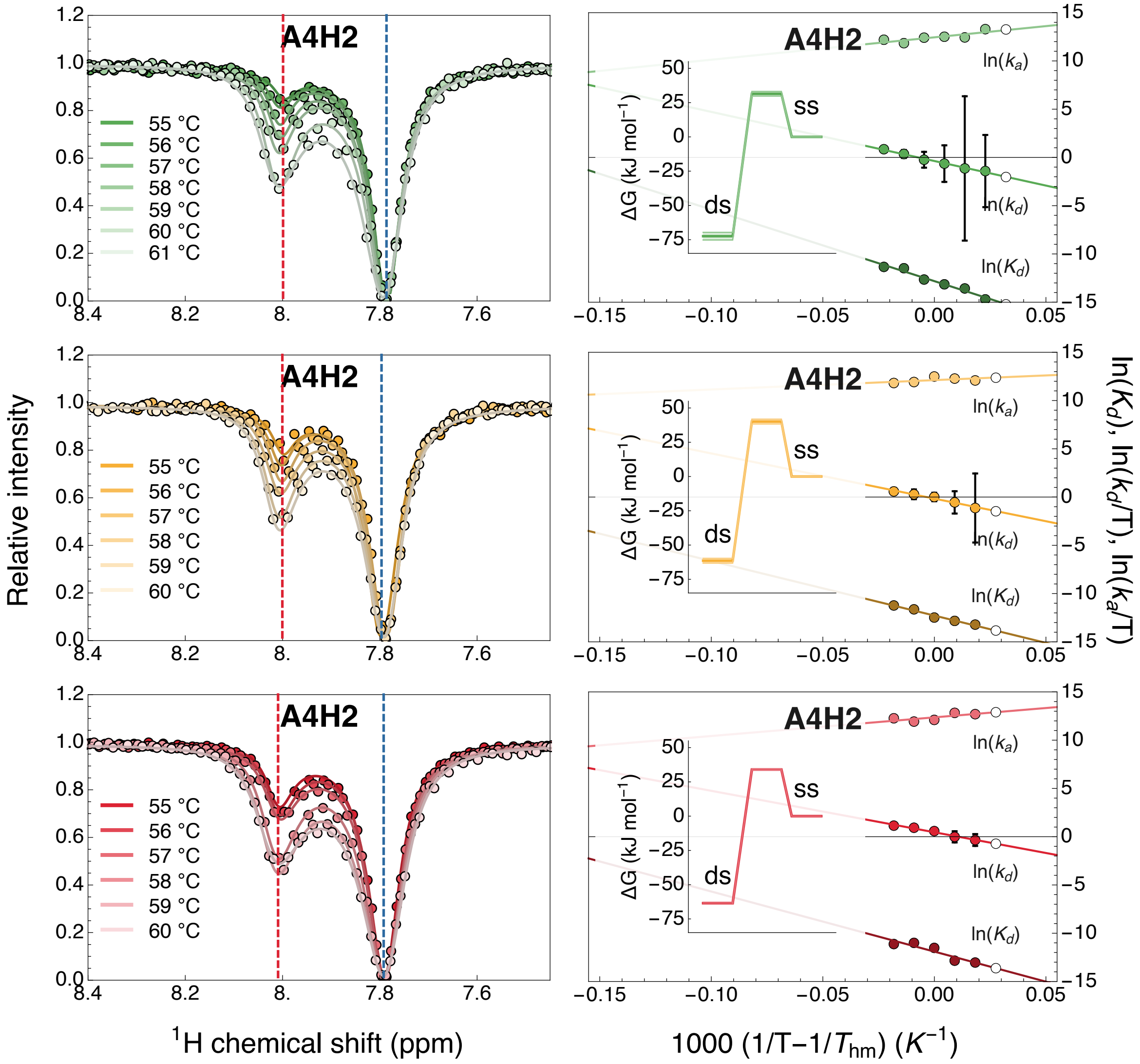

Figure S12: Temperature-dependent CEST melting profiles and van't Hoff plots for proton A4H2. $caC_{7.0}$, $caC_{5.8}$ and $caC_{4.7}$ are shown in shades of green, yellow and red, respectively. Dashed blue and red lines indicate GS and ES chemical shift values. In the van't Hoff plots, shades of green, yellow and red indicate data entries and linear fits for $caC_{7.0}$, $caC_{5.8}$ and $caC_{4.7}$, respectively. White data points represent back-calculated values. Insets present the relevant Gibbs free energy plots at each pH condition at 37 °C.

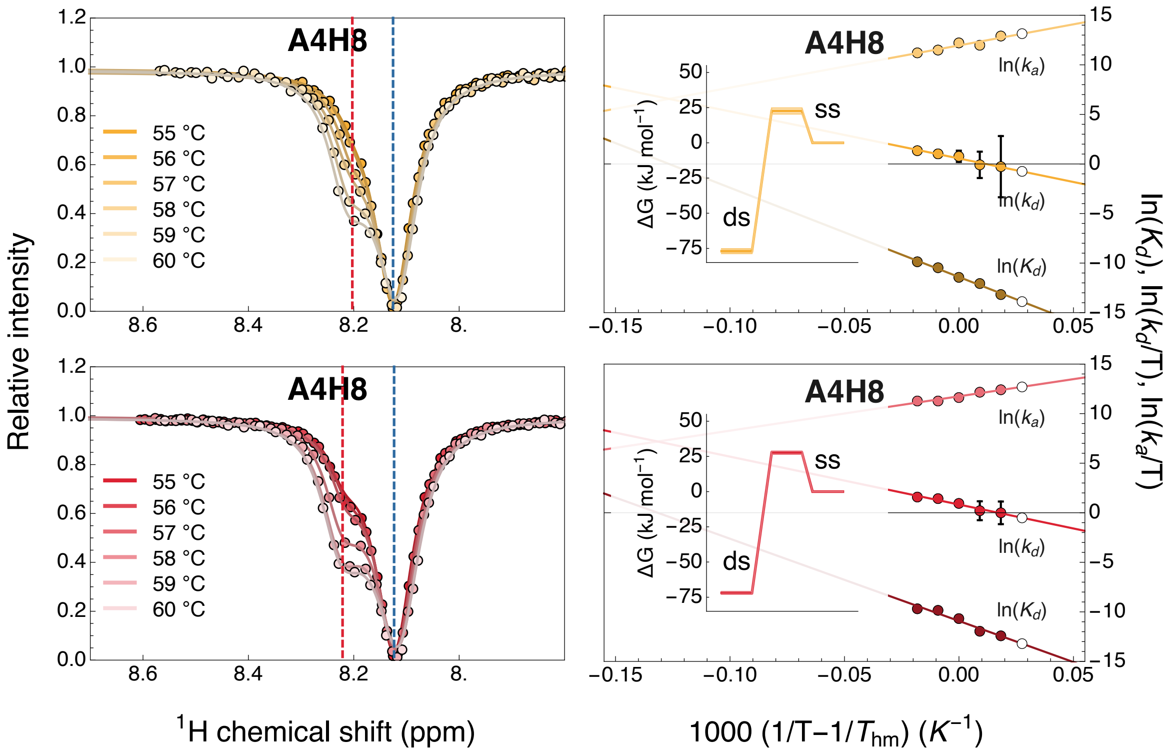

Figure S13: Temperature-dependent CEST melting profiles and van't Hoff plots for proton A4H8. caC$_{5.8}$ and caC$_{4.7}$ are shown in shades of yellow and red, respectively. Dashed blue and red lines indicate GS and ES chemical shift values. In the van't Hoff plots, shades of yellow and red indicate data entries and linear fits for caC$_{5.8}$ and caC$_{4.7}$, respectively. White data points represent back-calculated values. Insets present the relevant Gibbs free energy plots at each pH condition at 37 ℃.

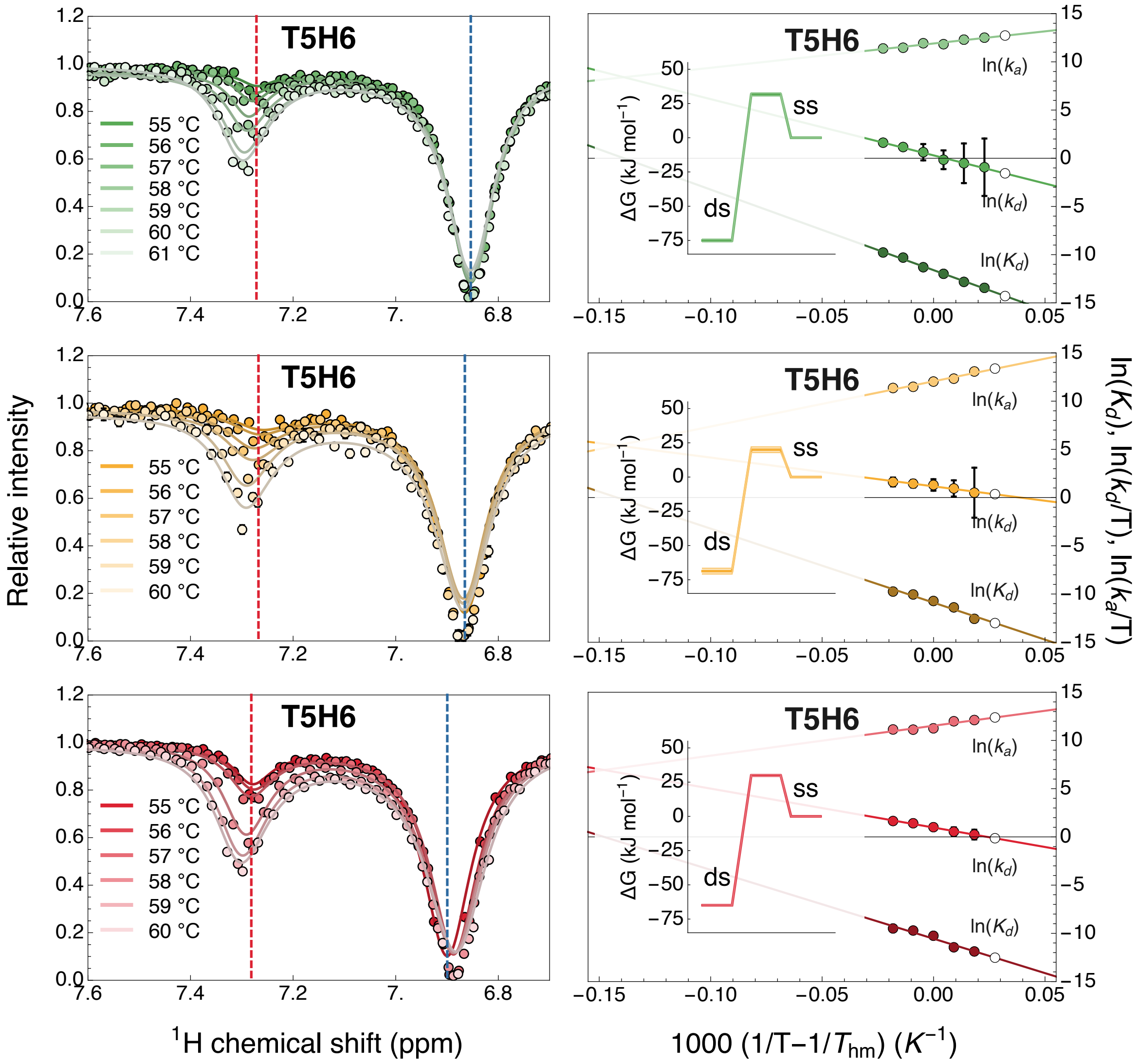

Figure S14: Temperature-dependent CEST melting profiles and van't Hoff plots for proton T5H6. caC$_{7.0}$, caC$_{5.8}$ and caC$_{4.7}$ are shown in shades of green, yellow and red, respectively. Dashed blue and red lines indicate GS and ES chemical shift values. In the van't Hoff plots, shades of green, yellow and red indicate data entries and linear fits for caC$_{7.0}$, caC$_{5.8}$ and caC$_{4.7}$, respectively. White data points represent back-calculated values. Insets present the relevant Gibbs free energy plots at each pH condition at 37 °C.

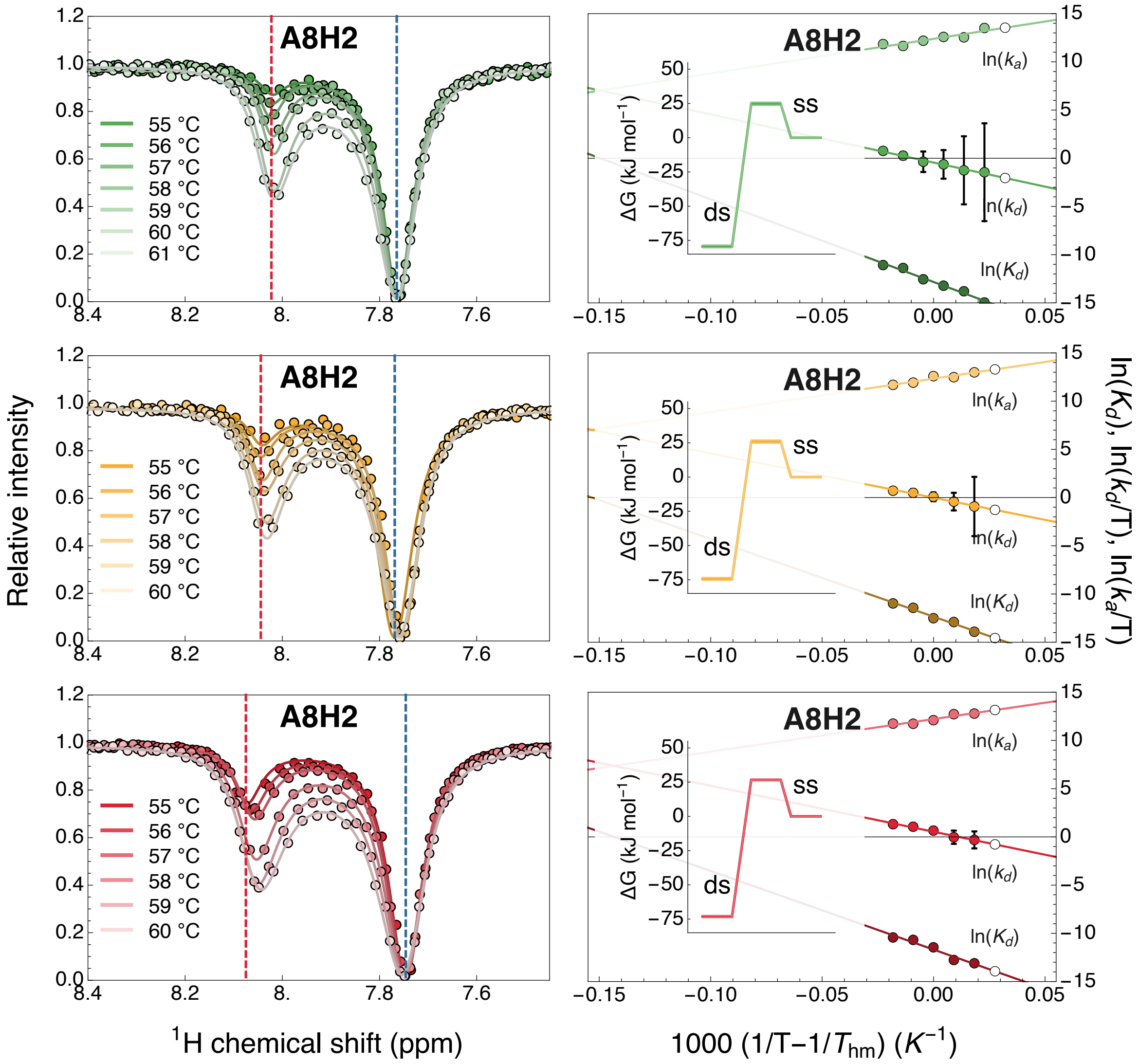

Figure S15: Temperature-dependent CEST melting profiles and van't Hoff plots for proton A8H2. caC$_{7.0}$, caC$_{5.8}$ and caC$_{4.7}$ are shown in shades of green, yellow and red, respectively. Dashed blue and red lines indicate GS and ES chemical shift values. In the van't Hoff plots, shades of green, yellow and red indicate data entries and linear fits for caC$_{7.0}$, caC$_{5.8}$ and caC$_{4.7}$, respectively. White data points represent back-calculated values. Insets present the relevant Gibbs free energy plots at each pH condition at 37 °C.

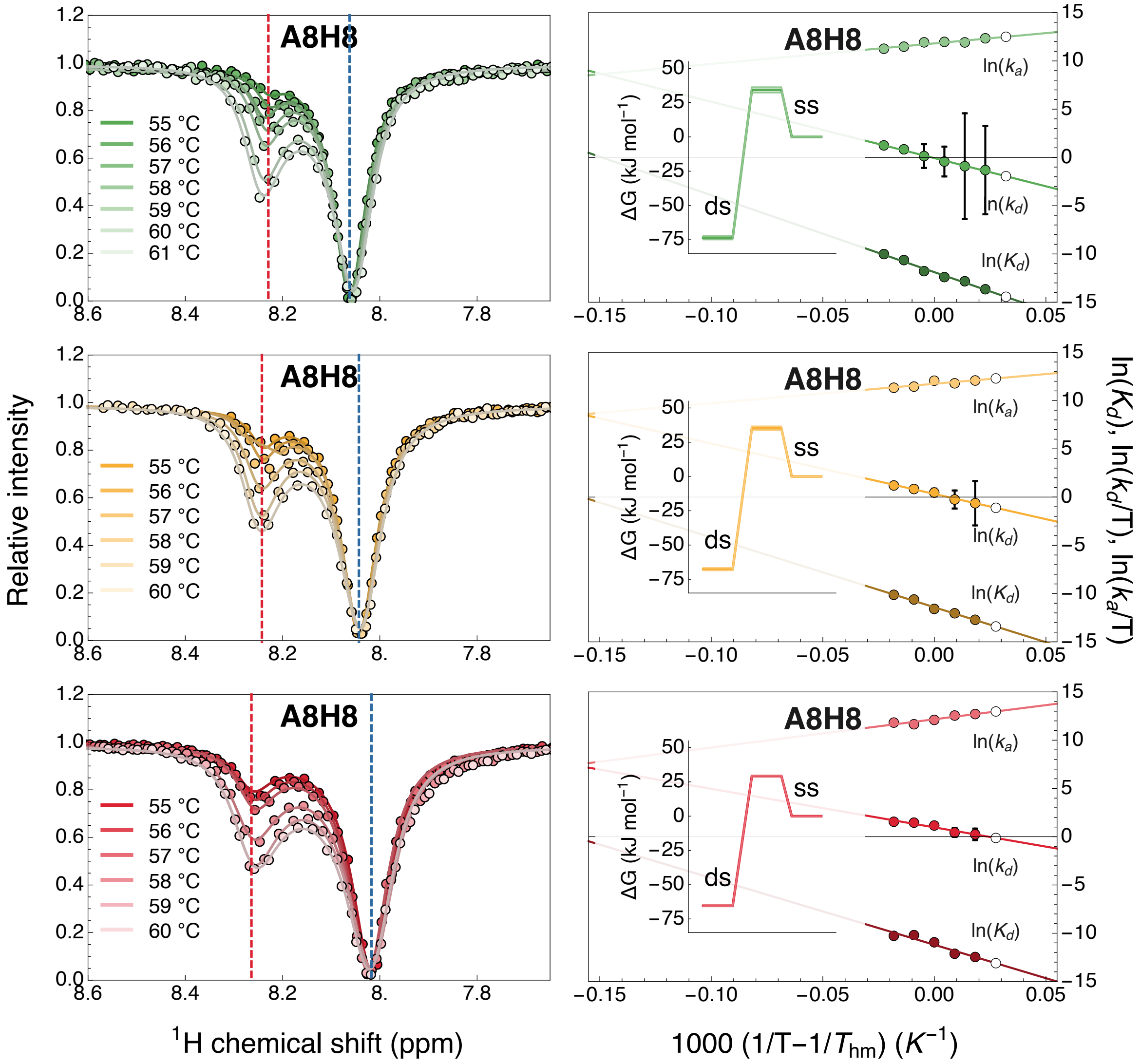

Figure S16: Temperature-dependent CEST melting profiles and van't Hoff plots for proton A8H8. $caC_{7.0}$, $caC_{5.8}$ and $caC_{4.7}$ are shown in shades of green, yellow and red, respectively. Dashed blue and red lines indicate GS and ES chemical shift values. In the van't Hoff plots, shades of green, yellow and red indicate data entries and linear fits for $caC_{7.0}$, $caC_{5.8}$ and $caC_{4.7}$, respectively. White data points represent back-calculated values. Insets present the relevant Gibbs free energy plots at each pH condition at 37 °C.

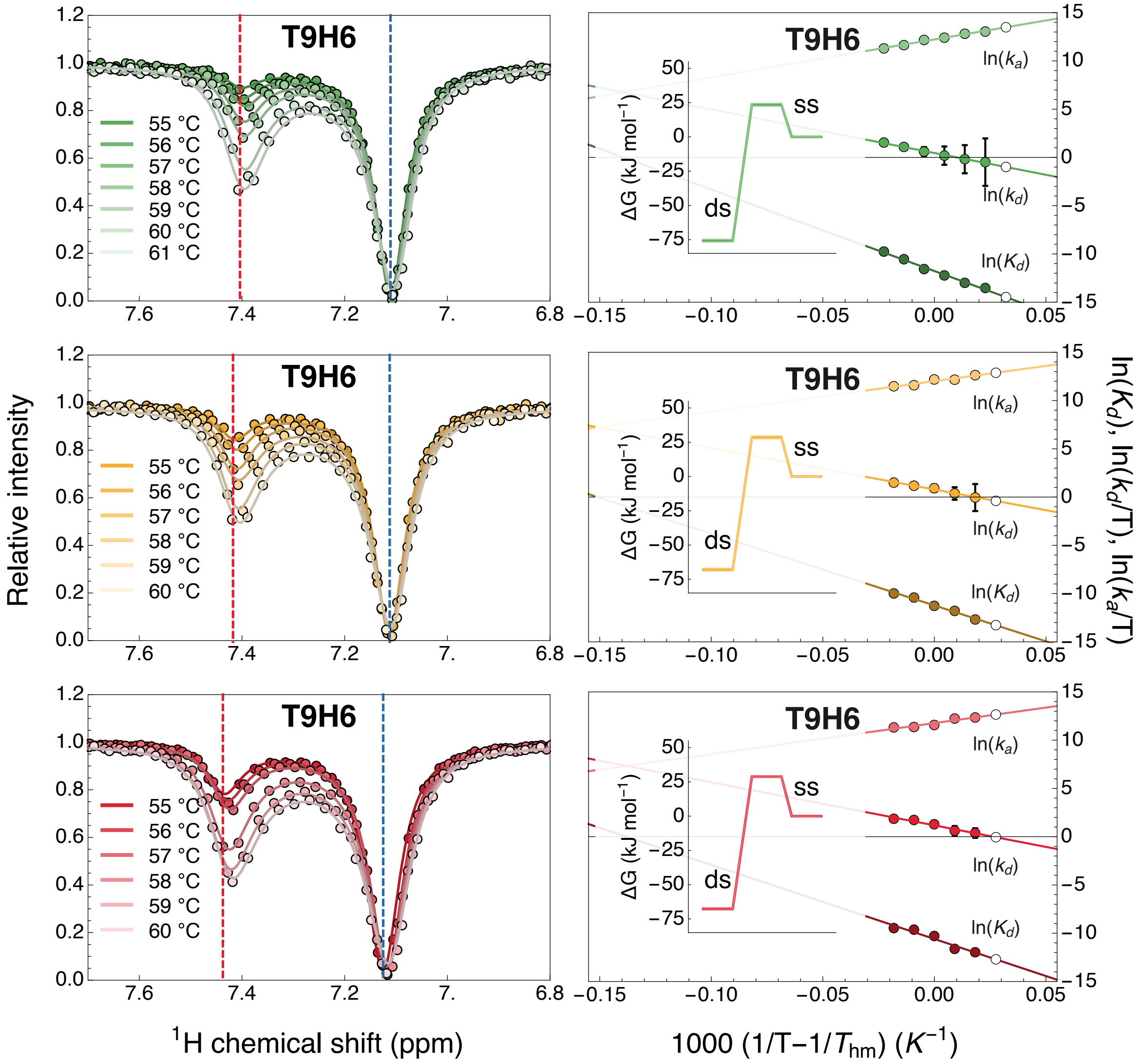

Figure S17: Temperature-dependent CEST melting profiles and van't Hoff plots for proton T9H6. $caC_{7.0}$, $caC_{5.8}$ and $caC_{4.7}$ are shown in shades of green, yellow and red, respectively. Dashed blue and red lines indicate GS and ES chemical shift values. In the van't Hoff plots, shades of green, yellow and red indicate data entries and linear fits for $caC_{7.0}$, $caC_{5.8}$ and $caC_{4.7}$, respectively. White data points represent back-calculated values. Insets present the relevant Gibbs free energy plots at each pH condition at 37 °C.

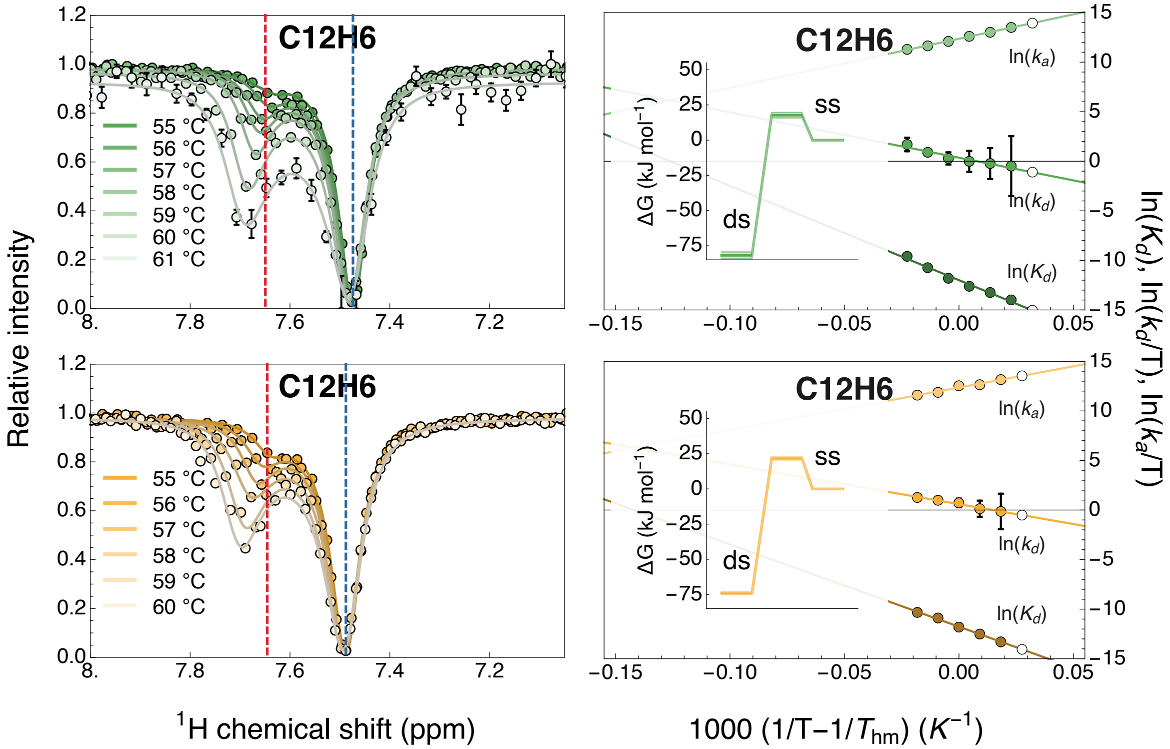

Figure S18: Temperature-dependent CEST melting profiles and van't Hoff plots for proton C12H6. caC$_{7.0}$, and caC$_{5.8}$ are shown in shades of green and yellow, respectively. Dashed blue and red lines indicate GS and ES chemical shift values. In the van't Hoff plots, shades of green and yellow indicate data entries and linear fits for caC$_{7.0}$ and caC$_{5.8}$, respectively. White data points represent back-calculated values. Insets present the relevant Gibbs free energy plots at each pH condition at 37 °C.

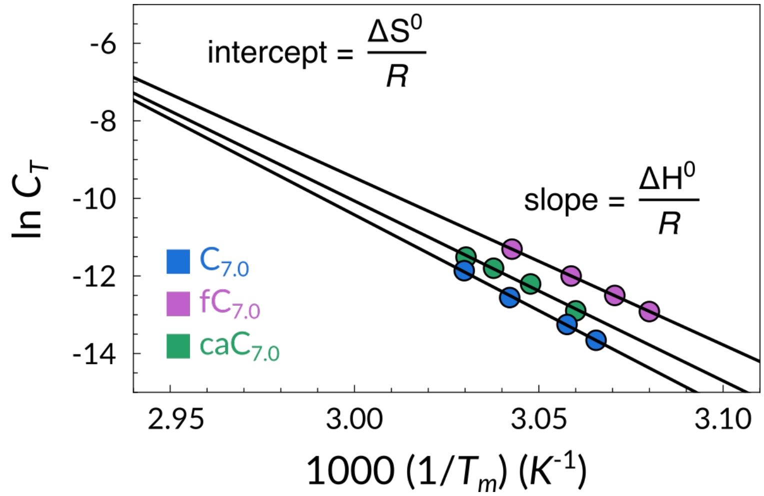

Figure S19: Concentration-dependent UV/Vis melting temperatures analysis of C$_{7.0}$ (blue), fC$_{7.0}$ (magenta) and caC$_{7.0}$ (green). The reciprocal of the observed T_m is plotted as a function of the logarithm of the total DNA concentration (C_t). The slope and the intercept of the fitted linear corresponds to $\Delta H^0/R$, and $\Delta S^0/R$, respectively.

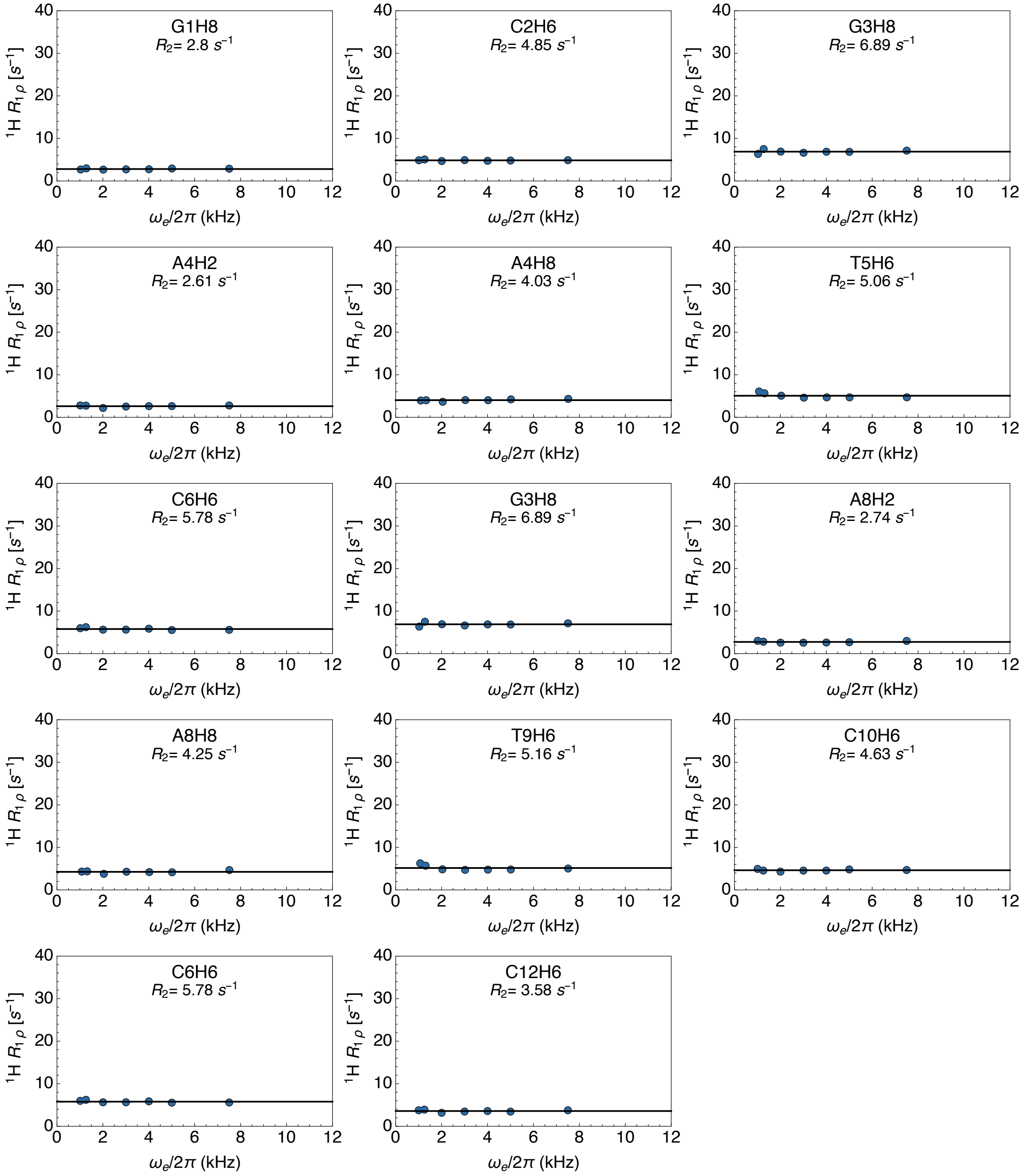

Figure S20: On-resonance $R_{1\rho}$ relaxation dispersion profiles for each available proton reported of $C_{7.0}$ recorded at 55 °C. Lines represent best fits either to models accounting for (color-coded) or discounting chemical exchange (black). Fitted R_2 relaxation rates are displayed for each site.

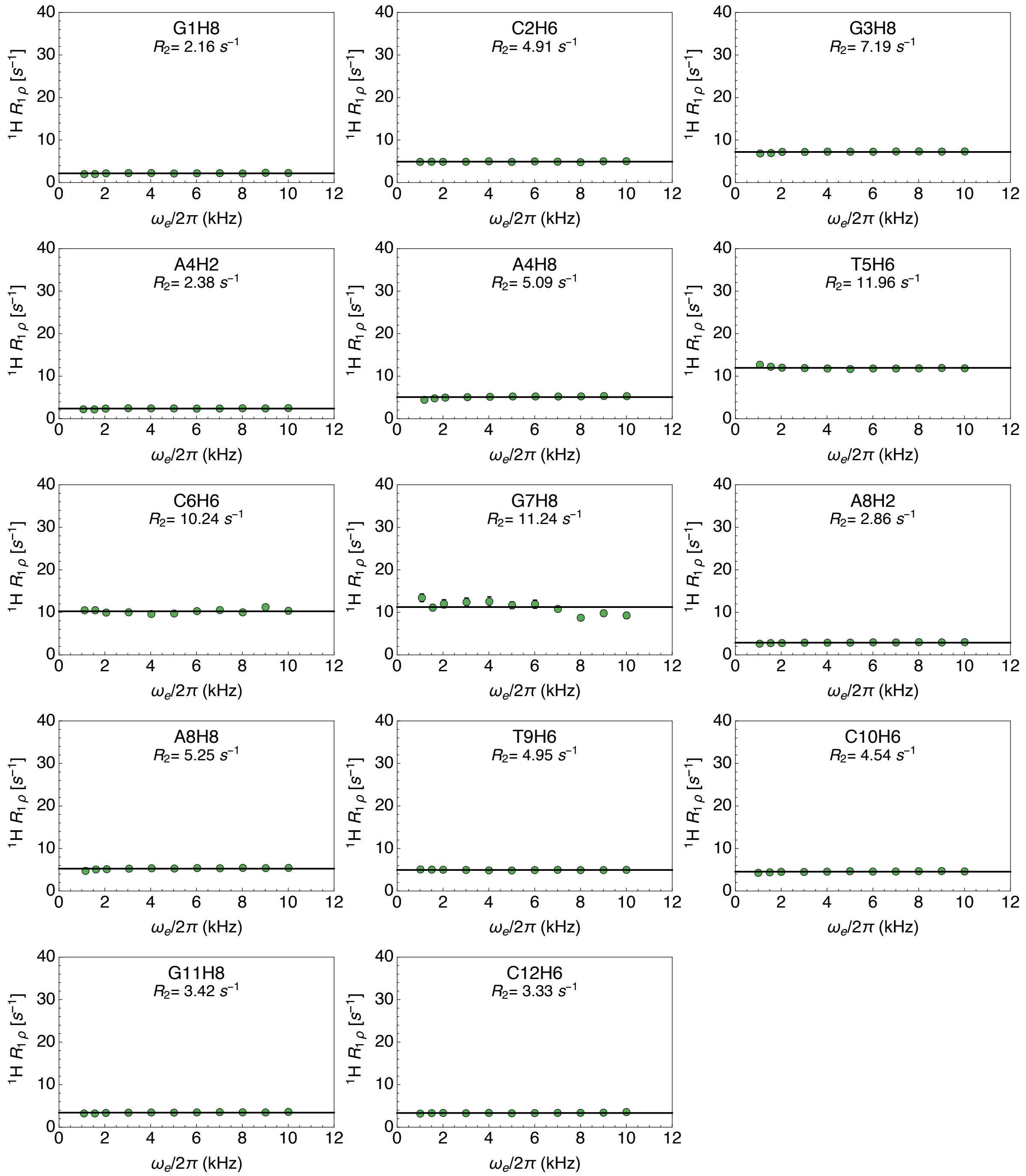

Figure S21: On-resonance $R_{1\rho}$ relaxation dispersion profiles for each available proton reported of caC$_{7.0}$ recorded at 55 ℃. Lines represent best fits either to models accounting for (color-coded) or discounting chemical exchange (black). Fitted R_2 relaxation rates are displayed for each site.

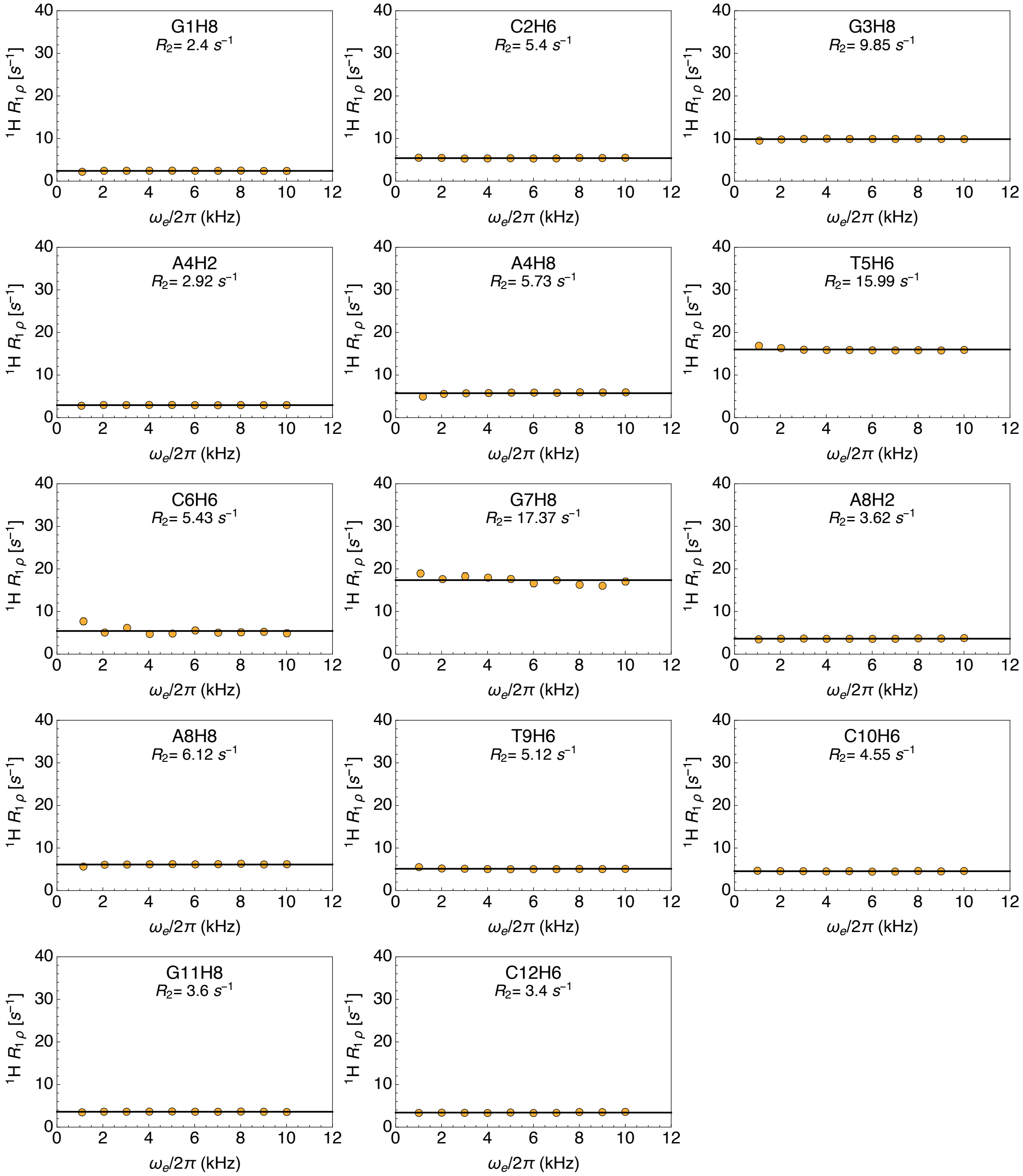

Figure S22: On-resonance $R_{1\rho}$ relaxation dispersion profiles for each available proton reported of $caC_{5.8}$ recorded at 55 °C. Lines represent best fits either to models accounting for (color-coded) or discounting chemical exchange (black). Fitted R_2 relaxation rates are displayed for each site.

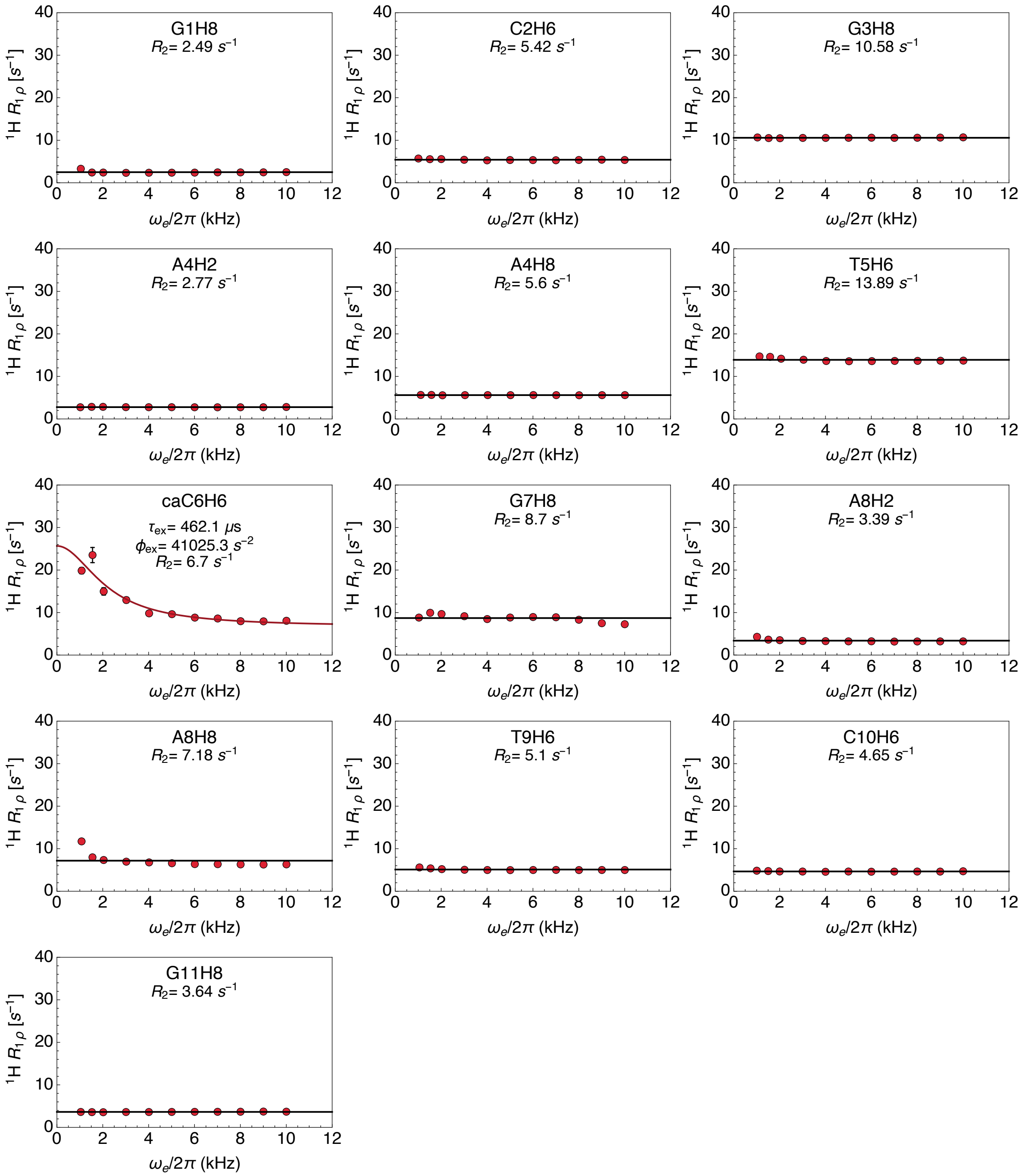

Figure S23: On-resonance $R_{1\rho}$ relaxation dispersion profiles for each available proton reported of $caC_{4.7}$ recorded at 55 °C. Lines represent best fits either to models accounting for (color-coded) or discounting chemical exchange (black). Fitted R_2 relaxation rates are displayed for each site, while exchange parameters such as τ_{ex} and ϕ_{ex} are only available for profiles best fit to the exchange model.

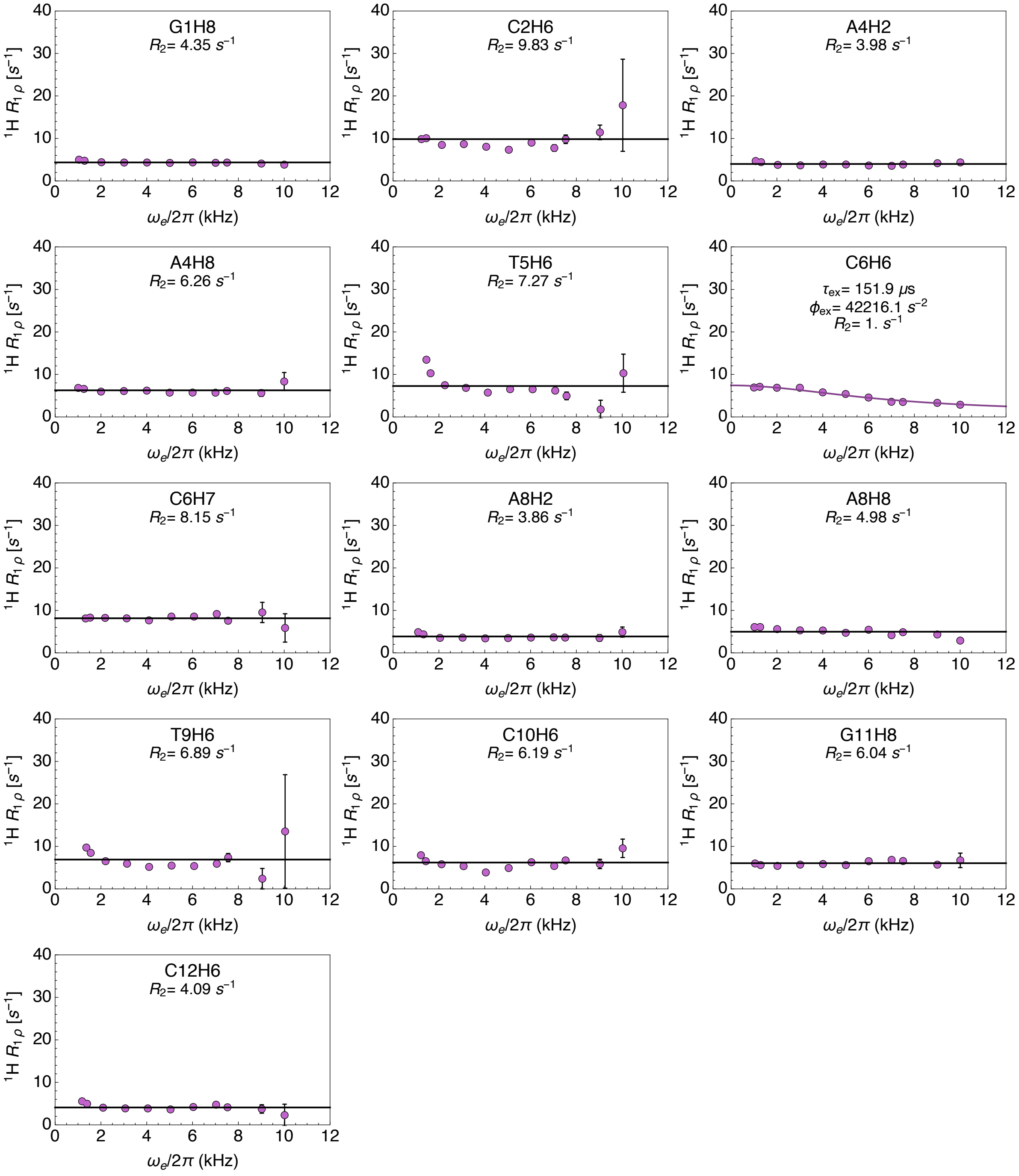

Figure S24: On-resonance $R_{1\rho}$ relaxation dispersion profiles for each available proton reported of fC$_{7.0}$ recorded at 55 °C. Lines represent best fits either to models accounting for (color-coded) or discounting chemical exchange (black). Fitted R_2 relaxation rates are displayed for each site, while exchange parameters such as τ_{ex} and ϕ_{ex} are only available for profiles best fit to the exchange model.

Table S1: Results of the fit of ^{1}H CEST measured at 800 MHz ^{1}H Larmor frequency. The temperature of each measurement is indicated on the leftmost column of the table. Chemical shifts relevant to dsDNA and ssDNA are given together with the relaxation parameters R_1 and R_2 for each available reporter of sample caC$_{7.0}$. Errors are given as one standard deviation.

		Chemical shift		Relaxation rates	
		δ(dsDNA) (ppm)	δ(ssDNA) (ppm)	R_1 (s^{-1})	R_2 (s^{-1})
56 ℃	C2H6	7.36 ± 2.17	7.462 ± 0.012	1.74 ± 0.02	4.82 ± 0.01
	A4H2	7.79 ± 0.44	8.008 ± 0.001	0.73 ± 0.02	1.94 ± 0.03
	T5H6	6.85 ± 0.37	7.276 ± 0.003	2.1 ± 0.02	11.51 ± 0.03
	A8H2	7.76 ± 0.62	8.025 ± 0.001	0.74 ± 0.02	2.2 ± 0.04
	A8H8	8.06 ± 0.27	8.229 ± 0.001	1.47 ± 0.02	5.08 ± 0.01
	T9H6	7.11 ± 0.29	7.402 ± 0.001	1.84 ± 0.03	4.86 ± 0.01
	C10H6	7.41 ± 0.18	7.557 ± 0.001	1.79 ± 0.03	4.47 ± 0.01
	C12H6	7.47 ± 0.27	7.649 ± 0.002	1.36 ± 0.02	3.17 ± 0.01
57 ℃	C2H6	7.36 ± 0.12	7.473 ± 0.002	1.67 ± 0.03	4.78 ± 0.04
	A4H2	7.79 ± 2.44	8.008 ± 0.004	0.73 ± 0.03	2.1 ± 0.07
	T5H6	6.86 ± 0.5	7.281 ± 0.002	2.08 ± 0.02	11.36 ± 0.03
	A8H2	7.76 ± 0.54	8.026 ± 0.001	0.76 ± 0.02	2.41 ± 0.05
	A8H8	8.06 ± 0.4	8.24 ± 0.002	1.44 ± 0.02	5.04 ± 0.01
	T9H6	7.11 ± 0.23	7.398 ± 0.001	1.77 ± 0.02	4.73 ± 0.01
	C10H6	7.4 ± 0.11	7.559 ± 0.001	1.73 ± 0.02	4.39 ± 0.01
	C12H6	7.48 ± 0.13	7.66 ± 0.002	1.31 ± 0.02	3.14 ± 0.01
58 ℃	C2H6	7.36 ± 2.32	7.467 ± 0.047	1.62 ± 0.04	4.57 ± 0.04
	A4H2	7.79 ± 0.14	8.008 ± 0.001	0.74 ± 0.02	2.24 ± 0.01
	T5H6	6.86 ± 0.24	7.288 ± 0.002	2.09 ± 0.03	10.95 ± 0.03
	A8H2	7.76 ± 0.21	8.022 ± 0.001	0.73 ± 0.03	2.22 ± 0.06
	A8H8	8.06 ± 0.1	8.235 ± 0.001	1.41 ± 0.02	4.96 ± 0.01
	T9H6	7.11 ± 0.12	7.4 ± 0.001	1.73 ± 0.02	4.63 ± 0.01
	C10H6	7.4 ± 0.08	7.561 ± 0.001	1.69 ± 0.02	4.29 ± 0.01
	C12H6	7.48 ± 0.1	7.665 ± 0.001	1.28 ± 0.02	3.1 ± 0.01

(*Continues on the next page...*)

59 ℃	C2H6	7.36 ± 0.03	7.485 ± 0.001	1.6 ± 0.02	4.4 ± 0.05
	A4H2	7.79 ± 0.13	8.011 ± 0.001	0.72 ± 0.03	2. ± 0.05
	T5H6	6.86 ± 0.18	7.293 ± 0.003	2.04 ± 0.04	10.69 ± 0.11
	A8H2	7.76 ± 0.17	8.02 ± 0.001	0.71 ± 0.02	2.21 ± 0.06
	A8H8	8.06 ± 0.21	8.238 ± 0.002	1.39 ± 0.02	4.71 ± 0.1
	T9H6	7.11 ± 0.06	7.399 ± 0.001	1.7 ± 0.02	4.75 ± 0.05
	C10H6	7.4 ± 0.05	7.566 ± 0.001	1.66 ± 0.03	4.29 ± 0.05
	C12H6	7.48 ± 0.05	7.673 ± 0.001	1.26 ± 0.02	3.1 ± 0.04
60 ℃	C2H6	7.36 ± 0.02	7.491 ± 0.001	1.52 ± 0.02	4.18 ± 0.02
	A4H2	7.79 ± 0.06	8.01 ± 0.001	0.74 ± 0.03	2.08 ± 0.02
	T5H6	6.87 ± 0.07	7.298 ± 0.003	2.19 ± 0.04	10.31 ± 0.06
	A8H2	7.77 ± 0.06	8.02 ± 0.001	0.72 ± 0.02	2.43 ± 0.02
	A8H8	8.06 ± 0.03	8.241 ± 0.001	1.33 ± 0.02	4.53 ± 0.02
	T9H6	7.12 ± 0.04	7.398 ± 0.001	1.58 ± 0.02	4.23 ± 0.02
	C10H6	7.41 ± 0.02	7.568 ± 0.001	1.58 ± 0.03	3.9 ± 0.02
	C12H6	7.49 ± 0.04	7.687 ± 0.001	1.21 ± 0.02	2.73 ± 0.02
61 ℃	C2H6	7.37 ± 0.07	7.498 ± 0.005	1.48 ± 0.03	4.59 ± 0.24
	A4H2	7.8 ± 0.06	8.014 ± 0.001	0.73 ± 0.02	2.52 ± 0.04
	T5H6	6.88 ± 0.03	7.301 ± 0.001	2.14 ± 0.04	10.77 ± 0.13
	A8H2	7.77 ± 0.06	8.021 ± 0.001	0.72 ± 0.02	2.62 ± 0.05
	A8H8	8.06 ± 0.02	8.244 ± 0.001	1.34 ± 0.02	4.86 ± 0.04
	T9H6	7.12 ± 0.02	7.4 ± 0.001	1.6 ± 0.02	4.59 ± 0.04
	C10H6	7.42 ± 0.12	7.578 ± 0.01	1.53 ± 0.12	4.3 ± 0.24
	C12H6	7.5 ± 0.1	7.693 ± 0.008	1.17 ± 0.03	2.92 ± 0.25

Table S2: Results of the fit of ^{1}H CEST measured at 800 MHz ^{1}H Larmor frequency. The temperature of each measurement is indicated on the leftmost column of the table. Chemical shifts relevant to dsDNA and ssDNA are given together with the relaxation parameters R_1 and R_2 for each available reporter of sample caC$_{5.8}$. Errors are given as one standard deviation.

		**Chemical shift**		**Relaxation rates**	
		δ(dsDNA) (ppm)	δ(ssDNA) (ppm)	R_1 (s^{-1})	R_2 (s^{-1})
56 ℃	C2H6	7.36 ± 0.11	7.48 ± 0.002	1.74 ± 0.025	5.23 ± 0.01
	A4H2	7.79 ± 0.38	8.005 ± 0.001	0.83 ± 0.015	2.64 ± 0.
	A4H8	8.12 ± 0.12	8.205 ± 0.002	1.53 ± 0.02	5.5 ± 0.01
	T5H6	6.87 ± 0.51	7.272 ± 0.004	2.67 ± 0.045	14.4 ± 0.05
	A8H2	7.75 ± 0.39	8.047 ± 0.001	0.82 ± 0.017	3.11 ± 0.02
	A8H8	8.04 ± 0.14	8.244 ± 0.001	1.48 ± 0.02	5.88 ± 0.01
	T9H6	7.11 ± 0.14	7.415 ± 0.001	1.81 ± 0.028	4.89 ± 0.01
	C10H6	7.41 ± 0.1	7.579 ± 0.001	1.76 ± 0.025	4.54 ± 0.01
	C12H6	7.49 ± 0.12	7.661 ± 0.002	1.35 ± 0.024	3.29 ± 0.01
57 ℃	C2H6	7.36 ± 0.05	7.486 ± 0.001	1.67 ± 0.03	5.08 ± 0.05
	A4H2	7.79 ± 0.11	8.005 ± 0.001	0.8 ± 0.02	2.22 ± 0.03
	A4H8	8.12 ± 0.03	8.207 ± 0.001	1.49 ± 0.02	5.51 ± 0.03
	T5H6	6.87 ± 0.17	7.281 ± 0.003	2.96 ± 0.05	15.21 ± 0.17
	A8H2	7.76 ± 0.14	8.042 ± 0.001	0.8 ± 0.01	2.52 ± 0.04
	A8H8	8.04 ± 0.07	8.244 ± 0.001	1.44 ± 0.02	5.66 ± 0.04
	T9H6	7.12 ± 0.1	7.413 ± 0.001	1.75 ± 0.02	4.86 ± 0.05
	C10H6	7.41 ± 0.06	7.582 ± 0.001	1.74 ± 0.03	4.48 ± 0.06
	C12H6	7.49 ± 0.07	7.67 ± 0.001	1.33 ± 0.02	3.18 ± 0.05
58 ℃	C2H6	7.36 ± 0.03	7.488 ± 0.001	1.59 ± 0.03	4.64 ± 0.04
	A4H2	7.79 ± 0.07	8.009 ± 0.001	0.76 ± 0.01	2.09 ± 0.04
	A4H8	8.12 ± 0.03	8.212 ± 0.001	1.45 ± 0.02	4.93 ± 0.04
	T5H6	6.88 ± 0.1	7.284 ± 0.003	2.91 ± 0.05	16.28 ± 0.28
	A8H2	7.76 ± 0.09	8.04 ± 0.001	0.76 ± 0.01	2.53 ± 0.05
	A8H8	8.04 ± 0.04	8.247 ± 0.001	1.38 ± 0.02	5.29 ± 0.05

(*Continues on the next page...*)

58 ℃	T9H6	7.12 ± 0.06	7.412 ± 0.001	1.71 ± 0.02	4.47 ± 0.05
	C10H6	7.41 ± 0.04	7.584 ± 0.001	1.65 ± 0.03	4.1 ± 0.05
	C12H6	7.49 ± 0.04	7.683 ± 0.001	1.27 ± 0.02	2.81 ± 0.04
59 ℃	C2H6	7.37 ± 0.02	7.492 ± 0.001	1.56 ± 0.03	5.01 ± 0.06
	A4H2	7.79 ± 0.06	8.009 ± 0.001	0.8 ± 0.01	2.49 ± 0.04
	A4H8	8.12 ± 0.02	8.213 ± 0.001	1.43 ± 0.02	5.44 ± 0.06
	T5H6	6.89 ± 0.08	7.295 ± 0.004	2.4 ± 0.06	14.47 ± 0.24
	A8H2	7.76 ± 0.07	8.036 ± 0.001	0.79 ± 0.02	2.93 ± 0.06
	A8H8	8.05 ± 0.02	8.248 ± 0.001	1.36 ± 0.02	5.84 ± 0.06
	T9H6	7.12 ± 0.04	7.41 ± 0.001	1.62 ± 0.02	4.81 ± 0.07
	C10H6	7.41 ± 0.03	7.585 ± 0.001	1.58 ± 0.03	4.39 ± 0.06
	C12H6	7.5 ± 0.04	7.693 ± 0.001	1.24 ± 0.02	2.96 ± 0.06
60 ℃	C2H6	7.37 ± 0.02	7.499 ± 0.001	1.5 ± 0.02	4.57 ± 0.06
	A4H2	7.8 ± 0.04	8.01 ± 0.001	0.79 ± 0.02	2.52 ± 0.05
	A4H8	8.12 ± 0.02	8.215 ± 0.001	1.41 ± 0.02	5.33 ± 0.07
	T5H6	6.9 ± 0.06	7.296 ± 0.004	1.98 ± 0.06	13.11 ± 0.21
	A8H2	7.77 ± 0.05	8.035 ± 0.001	0.77 ± 0.01	2.99 ± 0.06
	A8H8	8.05 ± 0.02	8.249 ± 0.001	1.32 ± 0.02	5.71 ± 0.06
	T9H6	7.13 ± 0.03	7.409 ± 0.002	1.57 ± 0.02	4.67 ± 0.06
	C10H6	7.41 ± 0.02	7.588 ± 0.001	1.53 ± 0.03	4.43 ± 0.06
	C12H6	7.5 ± 0.02	7.702 ± 0.001	1.19 ± 0.02	2.96 ± 0.06

Table S3: Results of the fit of ^{1}H CEST measured at 800 MHz ^{1}H Larmor frequency. The temperature of each measurement is indicated on the leftmost column of the table. Chemical shifts relevant to dsDNA and ssDNA are given together with the relaxation parameters R_1 and R_2 for each available reporter of sample caC$_{4.7}$. Errors are given as one standard deviation.

		Chemical shift		Relaxation rates	
		δ(dsDNA) (ppm)	δ(ssDNA) (ppm)	R_1 (s^{-1})	R_2 (s^{-1})
56 °C	C2H6	7.37 ± 0.06	7.496 ± 0.002	1.67 ± 0.03	5.21 ± 0.03
	A4H2	7.79 ± 0.08	8.008 ± 0.	0.82 ± 0.01	2.34 ± 0.03
	A4H8	8.12 ± 0.04	8.219 ± 0.001	1.5 ± 0.02	5.33 ± 0.03
	T5H6	6.89 ± 0.07	7.28 ± 0.001	2.22 ± 0.02	13.5 ± 0.06
	A8H2	7.75 ± 0.15	8.066 ± 0.001	0.81 ± 0.01	2.65 ± 0.04
	A8H8	8.02 ± 0.07	8.262 ± 0.001	1.49 ± 0.02	6.4 ± 0.04
	T9H6	7.12 ± 0.07	7.431 ± 0.001	1.81 ± 0.02	5.01 ± 0.04
	C10H6	7.41 ± 0.05	7.606 ± 0.001	1.76 ± 0.03	4.63 ± 0.04
57 °C	C2H6	7.37 ± 0.06	7.502 ± 0.002	1.64 ± 0.03	4.97 ± 0.04
	A4H2	7.79 ± 0.09	8.01 ± 0.001	0.81 ± 0.01	2.29 ± 0.04
	A4H8	8.12 ± 0.03	8.22 ± 0.001	1.48 ± 0.02	5.31 ± 0.03
	T5H6	6.89 ± 0.07	7.286 ± 0.001	2.22 ± 0.03	13.62 ± 0.06
	A8H2	7.75 ± 0.13	8.06 ± 0.001	0.8 ± 0.02	2.64 ± 0.05
	A8H8	8.03 ± 0.05	8.26 ± 0.001	1.42 ± 0.02	6. ± 0.04
	T9H6	7.12 ± 0.07	7.428 ± 0.001	1.77 ± 0.03	4.87 ± 0.04
	C10H6	7.41 ± 0.05	7.605 ± 0.001	1.76 ± 0.03	4.52 ± 0.04
58 °C	C2H6	7.38 ± 0.02	7.507 ± 0.001	1.5 ± 0.02	4.92 ± 0.05
	A4H2	7.79 ± 0.04	8.012 ± 0.001	0.79 ± 0.01	2.49 ± 0.03
	A4H8	8.12 ± 0.01	8.224 ± 0.	1.4 ± 0.02	5.11 ± 0.03
	T5H6	6.9 ± 0.03	7.294 ± 0.001	1.83 ± 0.02	13.18 ± 0.08
	A8H2	7.75 ± 0.05	8.057 ± 0.001	0.78 ± 0.01	2.73 ± 0.04
	A8H8	8.02 ± 0.04	8.264 ± 0.001	1.34 ± 0.02	6.24 ± 0.04
	T9H6	7.13 ± 0.03	7.428 ± 0.001	1.65 ± 0.02	4.77 ± 0.04
	C10H6	7.42 ± 0.02	7.609 ± 0.001	1.58 ± 0.02	4.4 ± 0.04

(*Continues on the next page...*)

59 ℃	C2H6	7.38 ± 0.02	7.514 ± 0.001	1.43 ± 0.02	4.75 ± 0.05
	A4H2	7.8 ± 0.03	8.013 ± 0.001	0.79 ± 0.01	2.74 ± 0.04
	A4H8	8.12 ± 0.01	8.225 ± 0.001	1.36 ± 0.02	4.97 ± 0.03
	T5H6	6.91 ± 0.02	7.301 ± 0.001	1.67 ± 0.02	12.85 ± 0.07
	A8H2	7.76 ± 0.04	8.054 ± 0.001	0.77 ± 0.02	2.88 ± 0.04
	A8H8	8.03 ± 0.02	8.262 ± 0.001	1.27 ± 0.02	6.4 ± 0.04
	T9H6	7.14 ± 0.02	7.426 ± 0.001	1.59 ± 0.02	4.44 ± 0.04
	C10H6	7.42 ± 0.02	7.61 ± 0.001	1.49 ± 0.02	4.19 ± 0.04
60 ℃	C2H6	7.38 ± 0.02	7.518 ± 0.001	1.37 ± 0.03	4.59 ± 0.02
	A4H2	7.8 ± 0.04	8.016 ± 0.001	0.8 ± 0.01	2.97 ± 0.01
	A4H8	8.12 ± 0.01	8.226 ± 0.001	1.32 ± 0.02	4.99 ± 0.01
	T5H6	6.92 ± 0.02	7.304 ± 0.002	1.61 ± 0.02	13.16 ± 0.04
	A8H2	7.76 ± 0.04	8.048 ± 0.001	0.76 ± 0.01	3.05 ± 0.01
	A8H8	8.03 ± 0.02	8.26 ± 0.001	1.14 ± 0.02	5.2 ± 0.02
	T9H6	7.14 ± 0.02	7.422 ± 0.002	1.48 ± 0.02	4.27 ± 0.01
	C10H6	7.42 ± 0.02	7.609 ± 0.001	1.37 ± 0.02	3.79 ± 0.02

Table S4: Results of the thermodynamic and kinetic analysis based on of ^{1}H CEST fitted data measured at 800 MHz ^{1}H Larmor frequency. The relevant temperature for each of the parameters' sets is indicated on the leftmost column of the table. The population of dsDNA, the kinetic rates and the dissociation equilibrium constants are given for each available reporter of sample caC$_{7.0}$. Errors are given as one standard deviation.

		**dsDNA population**	**Kinetic rates**				**Eq. constants**
		p_D (–)	k_{ex} (s^{-1})	k_d (s^{-1})	k_a (10^3 $M^{-1}s^{-1}$)	k_1 (s^{-1})	K_{eq} (10^{-6} M^{-1})
56 °C	C2H6	0.9652 ± 0.0249	28.36 ± 3.58	0.977 ± 0.694	635.849 ± 2014.48	27.387 ± 3.641	2.554 ± 2.693
	A4H2	0.9825 ± 0.0007	13.99 ± 0.88	0.245 ± 0.018	596.901 ± 45.487	13.75 ± 0.868	0.412 ± 0.032
	T5H6	0.9673 ± 0.0009	12.14 ± 1.1	0.397 ± 0.038	272.921 ± 25.826	11.747 ± 1.064	1.457 ± 0.085
	A8H2	0.9845 ± 0.0005	15.27 ± 1.26	0.237 ± 0.021	736.862 ± 67.056	15.03 ± 1.241	0.322 ± 0.023
	A8H8	0.9705 ± 0.0014	9.18 ± 1.26	0.271 ± 0.04	229.485 ± 33.069	8.912 ± 1.226	1.187 ± 0.117
	T9H6	0.9688 ± 0.0011	19.76 ± 1.54	0.617 ± 0.054	465.649 ± 38.815	19.142 ± 1.489	1.328 ± 0.095
	C10H6	0.9687 ± 0.0009	27.21 ± 2.38	0.852 ± 0.08	639.56 ± 58.795	26.356 ± 2.302	1.334 ± 0.082
	C12H6	0.9749 ± 0.0009	24.67 ± 1.67	0.619 ± 0.047	727.494 ± 55.507	24.046 ± 1.626	0.854 ± 0.062
57 °C	C2H6	0.958 ± 0.0019	26.28 ± 2.61	1.103 ± 0.118	456.048 ± 51.193	25.18 ± 2.503	2.431 ± 0.219
	A4H2	0.9695 ± 0.0114	10.61 ± 2.14	0.325 ± 0.146	333.599 ± 285.054	10.285 ± 2.064	1.446 ± 0.948
	T5H6	0.9559 ± 0.0027	13.45 ± 1.19	0.594 ± 0.068	221.954 ± 22.426	12.86 ± 1.137	2.692 ± 0.334
	A8H2	0.9724 ± 0.0015	10.33 ± 0.9	0.285 ± 0.029	277.255 ± 29.969	10.049 ± 0.879	1.035 ± 0.114
	A8H8	0.9549 ± 0.0045	9.15 ± 2.4	0.412 ± 0.115	148.669 ± 42.321	8.74 ± 2.288	2.831 ± 0.58
	T9H6	0.9595 ± 0.0012	20.5 ± 1.3	0.83 ± 0.061	368.712 ± 24.499	19.669 ± 1.239	2.255 ± 0.136
	C10H6	0.9562 ± 0.0014	24.68 ± 1.48	1.083 ± 0.079	408.743 ± 26.322	23.601 ± 1.415	2.653 ± 0.175
	C12H6	0.9638 ± 0.0009	21.87 ± 1.2	0.792 ± 0.049	442.122 ± 26.453	21.082 ± 1.16	1.793 ± 0.093
58 °C	C2H6	0.8951 ± 0.1962	25.58 ± 4.8	2.585 ± 5.082	-65.025 ± 2421.53	22.997 ± 7.102	93.137 ± 142.913
	A4H2	0.9622 ± 0.0009	13.91 ± 1.25	0.526 ± 0.048	269.025 ± 25.801	13.387 ± 1.207	1.957 ± 0.1
	T5H6	0.9333 ± 0.0027	12.97 ± 0.82	0.865 ± 0.069	137.934 ± 9.757	12.104 ± 0.76	6.288 ± 0.526
	A8H2	0.9635 ± 0.0011	14.67 ± 0.76	0.536 ± 0.033	293.994 ± 17.539	14.137 ± 0.728	1.826 ± 0.112
	A8H8	0.9457 ± 0.0018	11.92 ± 1.09	0.647 ± 0.062	157.797 ± 15.945	11.276 ± 1.037	4.113 ± 0.284
	T9H6	0.9406 ± 0.0014	20.32 ± 1.26	1.206 ± 0.081	244.39 ± 16.079	19.112 ± 1.187	4.941 ± 0.241
	C10H6	0.9399 ± 0.0016	21.3 ± 1.17	1.281 ± 0.079	252.837 ± 15.681	20.023 ± 1.104	5.075 ± 0.279
	C12H6	0.9513 ± 0.0013	20.25 ± 1.08	0.986 ± 0.06	300.413 ± 17.509	19.265 ± 1.027	3.286 ± 0.175
59 °C	C2H6	0.917 ± 0.0018	24.11 ± 1.11	2.001 ± 0.102	202.267 ± 10.274	22.112 ± 1.015	9.902 ± 0.441
	A4H2	0.952 ± 0.0015	16.41 ± 0.55	0.787 ± 0.034	247.313 ± 12.106	15.623 ± 0.524	3.191 ± 0.205
	T5H6	0.908 ± 0.0036	20.34 ± 1.32	1.872 ± 0.134	152.582 ± 12.567	18.472 ± 1.21	12.318 ± 1.007
	A8H2	0.9493 ± 0.0017	13.77 ± 0.69	0.698 ± 0.043	195.652 ± 11.632	13.068 ± 0.656	3.577 ± 0.238
	A8H8	0.9267 ± 0.0072	15.92 ± 1.39	1.168 ± 0.158	154.099 ± 20.951	14.749 ± 1.289	7.736 ± 1.554
	T9H6	0.9188 ± 0.0015	22.54 ± 0.78	1.831 ± 0.075	193.472 ± 7.373	20.706 ± 0.718	9.468 ± 0.357

(*Continues on the next page...*)

59 ℃	C10H6	0.9195 ± 0.0022	24.05 ± 0.98	1.935 ± 0.096	208.583 ± 10.249	22.11 ± 0.898	9.294 ± 0.535
	C12H6	0.9277 ± 0.0013	18.38 ± 0.8	1.329 ± 0.061	178.896 ± 8.83	17.049 ± 0.744	7.438 ± 0.286
60 ℃	C2H6	0.8642 ± 0.0031	21.9 ± 1.04	2.975 ± 0.159	105.764 ± 5.641	18.924 ± 0.902	28.158 ± 1.368
	A4H2	0.9156 ± 0.0026	16.88 ± 0.63	1.425 ± 0.07	138.985 ± 6.919	15.454 ± 0.578	10.277 ± 0.654
	T5H6	0.8522 ± 0.0034	21.62 ± 1.	3.195 ± 0.164	94.702 ± 5.234	18.429 ± 0.859	33.786 ± 1.705
	A8H2	0.9112 ± 0.0021	14.77 ± 0.54	1.311 ± 0.06	114.997 ± 4.952	13.454 ± 0.493	11.416 ± 0.571
	A8H8	0.8741 ± 0.0032	18.34 ± 0.81	2.308 ± 0.109	96.711 ± 5.381	16.031 ± 0.716	23.91 ± 1.292
	T9H6	0.8686 ± 0.0027	22.5 ± 0.89	2.956 ± 0.139	112.928 ± 4.929	19.545 ± 0.77	26.199 ± 1.16
	C10H6	0.868 ± 0.0029	22.96 ± 0.91	3.029 ± 0.134	114.639 ± 5.506	19.927 ± 0.798	26.458 ± 1.254
	C12H6	0.8795 ± 0.0028	20.41 ± 0.65	2.46 ± 0.097	113.092 ± 4.655	17.951 ± 0.57	21.777 ± 1.095
61 ℃	C2H6	0.8345 ± 0.0169	27.62 ± 5.47	4.591 ± 1.13	106.44 ± 22.406	23.024 ± 4.464	43.923 ± 9.729
	A4H2	0.9097 ± 0.0022	25.89 ± 0.56	2.338 ± 0.078	197.974 ± 6.55	23.551 ± 0.513	11.826 ± 0.598
	T5H6	0.8125 ± 0.0023	26.93 ± 0.75	5.05 ± 0.156	88.52 ± 2.756	21.876 ± 0.61	57.066 ± 1.56
	A8H2	0.8974 ± 0.0026	20.9 ± 0.43	2.144 ± 0.065	138.812 ± 5.201	18.754 ± 0.396	15.469 ± 0.819
	A8H8	0.8325 ± 0.0025	20.74 ± 0.5	3.473 ± 0.094	78.231 ± 2.417	17.264 ± 0.42	44.421 ± 1.44
	T9H6	0.8106 ± 0.0029	24.7 ± 0.48	4.678 ± 0.123	80.247 ± 2.036	20.024 ± 0.389	58.325 ± 1.987
	C10H6	0.7795 ± 0.0382	35.77 ± 7.03	7.918 ± 2.21	100.316 ± 35.853	27.853 ± 5.511	86.126 ± 30.785
	C12H6	0.7996 ± 0.0241	26.83 ± 3.43	5.374 ± 0.932	82.729 ± 16.851	21.453 ± 2.85	67.735 ± 18.616

Table S5: Results of the thermodynamic and kinetic analysis based on of ^{1}H CEST fitted data measured at 800 MHz [1]H Larmor frequency. The relevant temperature for each of the parameters' sets is indicated on the leftmost column of the table. The population of dsDNA, the kinetic rates and the dissociation equilibrium constants are given for each available reporter of sample $caC_{5.8}$. Errors are given as one standard deviation.

		**dsDNA population**	**Kinetic rates**				**Eq. constants**
		p_D (−)	k_{ex} (s^{-1})	k_d (s^{-1})	k_a (10^3 $M^{-1}s^{-1}$)	k_1 (s^{-1})	K_{eq} (10^{-6} M^{-1})
56 ℃	C2H6	0.9586 ± 0.0015	25.94 ± 2.35	1.075 ± 0.105	455.759 ± 44.699	24.861 ± 2.25	2.364 ± 0.169
	A4H2	0.9636 ± 0.0026	8.89 ± 1.27	0.322 ± 0.047	180.032 ± 31.536	8.564 ± 1.233	1.818 ± 0.265
	A4H8	0.963 ± 0.0018	20.71 ± 2.	0.767 ± 0.08	409.896 ± 46.152	19.946 ± 1.926	1.882 ± 0.186
	T5H6	0.9498 ± 0.0028	33.6 ± 4.24	1.687 ± 0.244	483.734 ± 63.779	31.914 ± 4.018	3.506 ± 0.401
	A8H2	0.974 ± 0.0009	15.23 ± 1.09	0.396 ± 0.031	433.742 ± 35.156	14.832 ± 1.06	0.914 ± 0.062
	A8H8	0.9535 ± 0.0016	11.34 ± 1.25	0.527 ± 0.062	176.776 ± 20.179	10.816 ± 1.187	2.988 ± 0.209
	T9H6	0.9528 ± 0.001	20.02 ± 1.45	0.946 ± 0.072	306.444 ± 23.03	19.076 ± 1.386	3.09 ± 0.127
	C10H6	0.9556 ± 0.001	25.59 ± 1.56	1.137 ± 0.075	417.699 ± 27.049	24.452 ± 1.494	2.725 ± 0.126
	C12H6	0.965 ± 0.0009	25.11 ± 1.74	0.879 ± 0.067	525.351 ± 38.026	24.227 ± 1.68	1.675 ± 0.086
57 ℃	C2H6	0.9308 ± 0.0019	21.11 ± 1.66	1.46 ± 0.12	215.67 ± 18.415	19.646 ± 1.55	6.782 ± 0.378
	A4H2	0.9559 ± 0.0011	13.19 ± 0.67	0.582 ± 0.035	217.205 ± 11.695	12.613 ± 0.637	2.681 ± 0.14
	A4H8	0.9359 ± 0.0014	14.24 ± 1.3	0.913 ± 0.084	157.915 ± 15.15	13.331 ± 1.216	5.789 ± 0.261
	T5H6	0.9115 ± 0.0033	29.17 ± 1.81	2.58 ± 0.164	228.542 ± 18.855	26.594 ± 1.674	11.338 ± 0.895
	A8H2	0.9575 ± 0.0009	15.58 ± 0.58	0.662 ± 0.029	266.515 ± 11.086	14.914 ± 0.552	2.485 ± 0.109
	A8H8	0.9352 ± 0.0017	12. ± 0.79	0.778 ± 0.057	131.466 ± 8.881	11.226 ± 0.733	5.926 ± 0.317
	T9H6	0.9269 ± 0.0016	19.95 ± 0.95	1.459 ± 0.079	192.037 ± 9.941	18.493 ± 0.882	7.602 ± 0.356
	C10H6	0.9335 ± 0.0016	25.57 ± 1.07	1.701 ± 0.079	272.579 ± 13.87	23.874 ± 1.003	6.248 ± 0.309
	C12H6	0.9483 ± 0.0009	22.09 ± 0.81	1.143 ± 0.049	307.314 ± 12.101	20.947 ± 0.769	3.721 ± 0.135
58 ℃	C2H6	0.9095 ± 0.0024	27.5 ± 1.21	2.489 ± 0.126	209.887 ± 11.376	25.014 ± 1.108	11.877 ± 0.649
	A4H2	0.9478 ± 0.001	19.29 ± 0.46	1.007 ± 0.032	265.775 ± 8.164	18.283 ± 0.439	3.793 ± 0.149
	A4H8	0.9138 ± 0.0023	24.93 ± 1.2	2.149 ± 0.116	200.719 ± 11.692	22.782 ± 1.104	10.724 ± 0.611
	T5H6	0.8789 ± 0.0043	30.35 ± 2.1	3.678 ± 0.304	167.271 ± 12.64	26.674 ± 1.836	22.038 ± 1.689
	A8H2	0.9484 ± 0.0009	21.12 ± 0.47	1.089 ± 0.034	294.777 ± 7.476	20.029 ± 0.44	3.696 ± 0.13
	A8H8	0.9198 ± 0.0014	19.81 ± 0.71	1.589 ± 0.061	172.334 ± 7.293	18.219 ± 0.658	9.231 ± 0.337
	T9H6	0.9065 ± 0.0021	26.73 ± 1.01	2.498 ± 0.111	196.801 ± 8.739	24.228 ± 0.915	12.705 ± 0.593
	C10H6	0.9085 ± 0.0019	28.22 ± 0.94	2.582 ± 0.099	212.718 ± 8.872	25.641 ± 0.858	12.153 ± 0.523
	C12H6	0.9276 ± 0.0013	28.43 ± 0.88	2.06 ± 0.071	276.303 ± 10.472	26.372 ± 0.817	7.46 ± 0.284
59 ℃	C2H6	0.8646 ± 0.0031	20.81 ± 1.18	2.817 ± 0.179	100.874 ± 6.092	17.99 ± 1.017	27.958 ± 1.392
	A4H2	0.9212 ± 0.0018	16.96 ± 0.65	1.337 ± 0.059	150.568 ± 6.987	15.627 ± 0.601	8.888 ± 0.425
	A4H8	0.864 ± 0.0042	20.34 ± 1.03	2.766 ± 0.158	98.167 ± 6.298	17.575 ± 0.9	28.252 ± 1.904

(*Continues on the next page...*)

59 ℃	T5H6	0.834 ± 0.0057	25.69 ± 1.78	4.267 ± 0.335	98.031 ± 7.735	21.427 ± 1.483	43.647 ± 3.256
	A8H2	0.9132 ± 0.0022	18.9 ± 0.69	1.641 ± 0.076	150.991 ± 6.546	17.261 ± 0.625	10.879 ± 0.572
	A8H8	0.8727 ± 0.0015	18.12 ± 0.67	2.307 ± 0.089	94.281 ± 3.729	15.815 ± 0.586	24.476 ± 0.621
	T9H6	0.8601 ± 0.0027	23.13 ± 1.02	3.235 ± 0.162	107.96 ± 5.162	19.893 ± 0.873	29.987 ± 1.267
	C10H6	0.8654 ± 0.0032	23.69 ± 0.96	3.189 ± 0.132	115.648 ± 6.297	20.503 ± 0.855	27.626 ± 1.415
	C12H6	0.8886 ± 0.0025	23.93 ± 0.69	2.665 ± 0.097	144.971 ± 5.468	21.265 ± 0.616	18.401 ± 0.86
60 ℃	C2H6	0.8346 ± 0.0036	27.2 ± 1.	4.5 ± 0.193	104.195 ± 4.7	22.704 ± 0.843	43.24 ± 2.073
	A4H2	0.9048 ± 0.0019	18.86 ± 0.58	1.795 ± 0.063	136.131 ± 5.405	17.067 ± 0.527	13.2 ± 0.568
	A4H8	0.8202 ± 0.0061	21.31 ± 1.17	3.831 ± 0.257	73.834 ± 4.823	17.474 ± 0.954	52.028 ± 3.937
	T5H6	0.8098 ± 0.0061	27.3 ± 2.47	5.195 ± 0.519	88.278 ± 8.334	22.109 ± 1.99	58.968 ± 4.268
	A8H2	0.8922 ± 0.0025	18.88 ± 0.63	2.036 ± 0.083	118.625 ± 4.984	16.847 ± 0.56	17.185 ± 0.861
	A8H8	0.8404 ± 0.0033	20.8 ± 0.93	3.318 ± 0.159	83.174 ± 4.375	17.48 ± 0.787	39.946 ± 1.823
	T9H6	0.8293 ± 0.0035	26.22 ± 0.82	4.476 ± 0.167	96.738 ± 3.863	21.749 ± 0.684	46.319 ± 2.13
	C10H6	0.8351 ± 0.0042	27.91 ± 0.94	4.603 ± 0.191	107.333 ± 4.898	23.31 ± 0.796	42.956 ± 2.396
	C12H6	0.8545 ± 0.0026	24.19 ± 0.87	3.52 ± 0.15	107.825 ± 4.132	20.672 ± 0.736	32.664 ± 1.244

Table S6: Results of the thermodynamic and kinetic analysis based on of ^{1}H CEST fitted data measured at 800 MHz ^{1}H Larmor frequency. The relevant temperature for each of the parameters' sets is indicated on the leftmost column of the table. The population of dsDNA, the kinetic rates and the dissociation equilibrium constants are given for each available reporter of sample caC$_{4.7}$. Errors are given as one standard deviation.

		**dsDNA population**	**Kinetic rates**				**Eq. constants**
		p_D (–)	k_{ex} (s^{-1})	k_d (s^{-1})	k_a (10^3 $M^{-1}s^{-1}$)	k_1 (s^{-1})	K_{eq} (10^{-6} M^{-1})
56 °C	C2H6	0.9392 ± 0.0016	24.26 ± 1.78	1.475 ± 0.115	284.68 ± 22.629	22.789 ± 1.677	5.189 ± 0.287
	A4H2	0.9602 ± 0.0006	17.92 ± 0.44	0.713 ± 0.022	328.342 ± 8.772	17.205 ± 0.419	2.171 ± 0.069
	A4H8	0.9462 ± 0.0011	18.42 ± 1.25	0.992 ± 0.068	245.787 ± 17.88	17.431 ± 1.183	4.039 ± 0.167
	T5H6	0.93 ± 0.0008	18.28 ± 0.54	1.28 ± 0.041	184.298 ± 5.944	17. ± 0.506	6.945 ± 0.171
	A8H2	0.9613 ± 0.0007	19.05 ± 0.64	0.736 ± 0.027	359.525 ± 14.06	18.31 ± 0.612	2.049 ± 0.072
	A8H8	0.9474 ± 0.0009	24.13 ± 0.68	1.271 ± 0.044	329.571 ± 10.293	22.862 ± 0.641	3.857 ± 0.135
	T9H6	0.9332 ± 0.001	21.86 ± 0.74	1.46 ± 0.051	231.693 ± 8.922	20.398 ± 0.69	6.307 ± 0.188
	C10H6	0.9348 ± 0.001	24.84 ± 0.96	1.62 ± 0.068	270.121 ± 11.293	23.219 ± 0.9	6.002 ± 0.195
57 °C	C2H6	0.9261 ± 0.0026	25.92 ± 1.4	1.914 ± 0.12	246.919 ± 16.586	24.003 ± 1.303	7.774 ± 0.554
	A4H2	0.9563 ± 0.0008	22.53 ± 0.63	0.985 ± 0.034	374.083 ± 12.66	21.547 ± 0.603	2.635 ± 0.104
	A4H8	0.9325 ± 0.0013	18.41 ± 1.17	1.243 ± 0.083	193.036 ± 12.685	17.167 ± 1.089	6.441 ± 0.252
	T5H6	0.914 ± 0.0014	20.02 ± 0.82	1.722 ± 0.077	161.46 ± 7.203	18.298 ± 0.754	10.671 ± 0.364
	A8H2	0.9545 ± 0.0009	21.29 ± 0.55	0.968 ± 0.03	339.092 ± 11.859	20.317 ± 0.529	2.858 ± 0.12
	A8H8	0.9379 ± 0.0009	24.8 ± 0.66	1.539 ± 0.047	284.431 ± 8.727	23.258 ± 0.615	5.413 ± 0.164
	T9H6	0.9208 ± 0.0014	23.46 ± 0.97	1.857 ± 0.081	207.039 ± 9.648	21.599 ± 0.895	8.977 ± 0.326
	C10H6	0.9234 ± 0.0015	29.96 ± 1.15	2.294 ± 0.102	274.297 ± 11.575	27.667 ± 1.062	8.368 ± 0.343
58 °C	C2H6	0.872 ± 0.0022	22.3 ± 0.81	2.856 ± 0.111	115.282 ± 4.869	19.448 ± 0.709	24.792 ± 0.915
	A4H2	0.917 ± 0.0014	21.5 ± 0.32	1.785 ± 0.039	180.22 ± 4.521	19.716 ± 0.301	9.913 ± 0.355
	A4H8	0.8764 ± 0.002	20.67 ± 0.7	2.556 ± 0.102	111.197 ± 4.036	18.116 ± 0.611	22.997 ± 0.814
	T5H6	0.8492 ± 0.002	18.15 ± 0.51	2.737 ± 0.09	77.547 ± 2.308	15.412 ± 0.428	35.309 ± 1.014
	A8H2	0.914 ± 0.0013	22.38 ± 0.45	1.925 ± 0.049	180.554 ± 4.705	20.459 ± 0.41	10.667 ± 0.345
	A8H8	0.8907 ± 0.002	29.11 ± 0.77	3.18 ± 0.107	180.118 ± 5.762	25.927 ± 0.68	17.669 ± 0.699
	T9H6	0.8518 ± 0.0024	24.37 ± 0.57	3.612 ± 0.096	106.331 ± 3.394	20.763 ± 0.497	33.997 ± 1.184
	C10H6	0.8685 ± 0.0021	28.54 ± 0.81	3.754 ± 0.119	143.003 ± 4.943	24.786 ± 0.706	26.271 ± 0.893
59 °C	C2H6	0.8287 ± 0.0041	26.31 ± 1.01	4.505 ± 0.204	96.638 ± 4.609	21.8 ± 0.841	46.691 ± 2.459
	A4H2	0.8935 ± 0.0017	23.69 ± 0.51	2.523 ± 0.071	150.858 ± 4.054	21.168 ± 0.457	16.734 ± 0.575
	A4H8	0.8191 ± 0.0041	22.98 ± 0.73	4.158 ± 0.147	79.013 ± 3.655	18.826 ± 0.623	52.709 ± 2.665
	T5H6	0.807 ± 0.0024	21.37 ± 0.63	4.125 ± 0.133	67.812 ± 2.227	17.248 ± 0.507	60.86 ± 1.689
	A8H2	0.8761 ± 0.0024	23.26 ± 0.51	2.882 ± 0.085	124.813 ± 3.949	20.374 ± 0.452	23.109 ± 0.977
	A8H8	0.8457 ± 0.0021	27.76 ± 0.76	4.282 ± 0.134	115.458 ± 3.565	23.473 ± 0.644	37.105 ± 1.096

(*Continues on the next page...*)

59 ℃	T9H6	0.8005 ± 0.0029	28.46 ± 0.57	5.678 ± 0.144	86.667 ± 2.27	22.782 ± 0.459	65.546 ± 2.127
	C10H6	0.834 ± 0.0031	31.81 ± 1.03	5.28 ± 0.177	121.359 ± 5.283	26.535 ± 0.89	43.561 ± 1.797
60 ℃	C2H6	0.8323 ± 0.0039	31.1 ± 0.86	5.216 ± 0.191	117.175 ± 4.576	25.885 ± 0.724	44.581 ± 2.305
	A4H2	0.8998 ± 0.0018	31.34 ± 0.52	3.14 ± 0.079	213.587 ± 5.433	28.198 ± 0.465	14.713 ± 0.564
	A4H8	0.8052 ± 0.0049	25.08 ± 0.71	4.886 ± 0.17	78.74 ± 3.559	20.198 ± 0.603	62.173 ± 3.523
	T5H6	0.7877 ± 0.0027	24.53 ± 0.59	5.208 ± 0.143	69.072 ± 1.993	19.324 ± 0.467	75.442 ± 2.221
	A8H2	0.8607 ± 0.0029	27.09 ± 0.69	3.774 ± 0.125	127.096 ± 4.477	23.32 ± 0.597	29.724 ± 1.363
	A8H8	0.8508 ± 0.0023	31.44 ± 0.58	4.691 ± 0.111	136.03 ± 3.623	26.746 ± 0.502	34.507 ± 1.172
	T9H6	0.7852 ± 0.0038	29.81 ± 0.51	6.405 ± 0.172	82.693 ± 2.145	23.408 ± 0.396	77.523 ± 3.164
	C10H6	0.8392 ± 0.0031	38.61 ± 0.69	6.21 ± 0.163	152.914 ± 4.437	32.401 ± 0.589	40.652 ± 1.734

Table S7: Thermodynamic and kinetic parameters of the dsDNA melting process obtained from the van't Hoff and Eyring analysis of the CEST-derived exchange parameters. Errors are given as one standard deviation.

		**Thermodynamics**				**Dissociation kinetics**			**Association kinetics**		
		$\Delta G^0_{37\mathbf{C}}$ kJ mol^{-1}	ΔH^0 kJ mol^{-1}	ΔS^0 J K^{-1} mol^{-1}	T_m (1 mM) ℃	$\Delta G^\ddagger_{d,37\mathbf{C}}$ kJ mol^{-1}	$\Delta H^\ddagger_d$ kJ mol^{-1}	$\Delta S^\ddagger_d$ J K^{-1} mol^{-1}	$\Delta G^\ddagger_{a,37\mathbf{C}}$ kJ mol^{-1}	$\Delta H^\ddagger_a$ kJ mol^{-1}	$\Delta S^\ddagger_a$ J K^{-1} mol^{-1}
	C2H6	71.1 ± 1.7	641 ± 26	1838 ± 77	65 ± 0.2	99.2 ± 2.2	372 ± 31	880 ± 94	28.1 ± 2.1	-269 ± 30	-958 ± 91
	G3H8	69. ± 1.3	608 ± 20	1738 ± 59	66 ± 0.2	106.4 ± 1.9	459 ± 28	1138 ± 84	37.4 ± 1.6	-149 ± 23	-600 ± 71
	A4H2	72.5 ± 0.9	626 ± 14	1784 ± 41	67 ± 0.2	100.1 ± 0.7	363 ± 11	847 ± 32	27.7 ± 0.7	-263 ± 11	-936 ± 32
	A4H8	77.6 ± 1.1	732 ± 16	2110 ± 48	65 ± 0.1	108.3 ± 1.8	498 ± 25	1258 ± 76	30.7 ± 1.8	-234 ± 25	-852 ± 75
	T5H6	71. ± 1.6	633 ± 23	1812 ± 70	65 ± 0.2	102.7 ± 1.4	415 ± 20	1006 ± 60	31.7 ± 1.3	-218 ± 19	-806 ± 57
$C_{7.0}$	C6H6	70.7 ± 1.4	638 ± 21	1829 ± 63	65 ± 0.2	99.8 ± 1.2	379 ± 17	900 ± 51	29.1 ± 1.2	-259 ± 17	-929 ± 51
	A8H2	73.8 ± 1.1	643 ± 17	1834 ± 50	67 ± 0.2	103.6 ± 1.	409 ± 15	986 ± 45	29.8 ± 1.	-233 ± 14	-849 ± 42
	A8H8	73.1 ± 1.	666 ± 15	1910 ± 45	65 ± 0.1	104.3 ± 1.	439 ± 14	1078 ± 43	31.2 ± 0.9	-227 ± 13	-833 ± 40
	T9H6	70.2 ± 1.8	626 ± 27	1791 ± 80	65 ± 0.3	102.2 ± 1.5	410 ± 22	993 ± 66	31.9 ± 1.5	-216 ± 22	-798 ± 65
	C10H6	71.3 ± 1.4	642 ± 21	1841 ± 64	65 ± 0.2	100.3 ± 1.3	382 ± 19	908 ± 56	29. ± 1.3	-260 ± 20	-933 ± 59
	C12H6	77.6 ± 1.1	723 ± 17	2082 ± 50	65 ± 0.1	104.2 ± 1.3	434 ± 18	1064 ± 55	26.6 ± 1.3	-289 ± 19	-1018 ± 58
	UV/Vis	55.2 ± 2.5	408 ± 43	1130 ± 131	68.8 ± 1.5						
	C2H6	73.3 ± 7.4	669 ± 106	1920 ± 318	65.2 ± 1.	93. ± 4.3	287 ± 64	625 ± 192	19.7 ± 4.1	-382 ± 61	-1295 ± 183
	A4H2	72.4 ± 2.8	607 ± 38	1725 ± 113	67.7 ± 0.4	103.9 ± 1.7	412 ± 24	993 ± 70	31.5 ± 1.7	-195 ± 24	-731 ± 71
	T5H6	75. ± 0.9	696 ± 14	2002 ± 42	64.8 ± 0.1	106.6 ± 1.1	481 ± 16	1208 ± 49	31.6 ± 1.1	-215 ± 16	-794 ± 48
	A8H2	79.4 ± 1.0	715 ± 14	2048 ± 44	66.2 ± 0.2	104.2 ± 1.1	414 ± 16	998 ± 47	24.8 ± 1.1	-301 ± 17	-1050 ± 50
$caC_{7.0}$	A8H8	73.6 ± 1.4	663 ± 21	1899 ± 63	65.5 ± 0.2	107.9 ± 2.	485 ± 29	1217 ± 88	34.3 ± 2.1	-177 ± 30	-682 ± 91
	T9H6	75.7 ± 0.8	700 ± 13	2014 ± 38	65. ± 0.1	99.2 ± 0.9	372 ± 13	879 ± 40	23.4 ± 0.9	-328 ± 13	-1134 ± 40
	C10H6	77.3 ± 3.6	727 ± 59	2096 ± 180	64.7 ± 0.6	98.6 ± 3.2	371 ± 51	879 ± 156	21.3 ± 2.9	-356 ± 46	-1217 ± 140
	C12H6	81.8 ± 2.4	785 ± 38	2267 ± 114	64.6 ± 0.3	99.6 ± 1.7	374 ± 26	884 ± 80	17.7 ± 1.9	-411 ± 30	-1382 ± 91
	UV/Vis	52.9 ± 2.9	388 ± 52	1079.8 ± 158	67.7 ± 1.7						
	C2H6	71.1 ± 1.2	660 ± 19	1899 ± 56	64.2 ± 0.2	94.8 ± 1.4	324 ± 22	739 ± 67	23.7 ± 1.3	-336 ± 19	-1160 ± 58
	A4H2	61.4 ± 1.7	470 ± 26	1316 ± 80	68.8 ± 0.6	101.5 ± 1.8	392 ± 28	937 ± 84	40.1 ± 1.7	-78 ± 26	-379 ± 79
	A4H8	77. ± 1.7	750 ± 26	2170 ± 77	63.6 ± 0.2	99.6 ± 1.8	391 ± 28	939 ± 84	22.6 ± 2.1	-359 ± 32	-1231 ± 97
	T5H6	68.7 ± 2.1	641 ± 32	1844 ± 97	63.8 ± 0.3	88.7 ± 1.9	244 ± 30	501 ± 89	20. ± 2.	-397 ± 32	-1343 ± 95
$caC_{5.8}$	A8H2	74.4 ± 1.1	671 ± 17	1923 ± 51	65.6 ± 0.2	100.3 ± 1.2	379 ± 18	897 ± 54	25.9 ± 1.2	-292 ± 18	-1026 ± 55
	A8H8	67.6 ± 1.	602 ± 15	1722 ± 47	65. ± 0.2	102.8 ± 1.4	432 ± 21	1062 ± 64	35.2 ± 1.5	-170 ± 23	-660 ± 69
	T9H6	68. ± 0.9	615 ± 14	1765 ± 43	64.5 ± 0.2	96.6 ± 0.9	352 ± 13	825 ± 40	28.6 ± 1.	-263 ± 15	-940 ± 45
	C10H6	69.7 ± 1.	639 ± 16	1834 ± 50	64.4 ± 0.2	93.6 ± 1.	309 ± 16	694 ± 47	23.9 ± 1.1	-330 ± 17	-1140 ± 51
	C12H6	74.1 ± 0.9	690 ± 14	1985 ± 42	64.6 ± 0.1	95.7 ± 1.	331 ± 16	760 ± 48	21.6 ± 1.	-358 ± 16	-1225 ± 47
	C2H6	58.6 ± 0.7	483 ± 11	1369 ± 32	65.6 ± 0.2	93.1 ± 0.7	309 ± 11	695 ± 34	34.5 ± 0.9	-175 ± 14	-674 ± 42
	A4H2	58.5 ± 0.5	441 ± 8	1232 ± 24	68.5 ± 0.2	96.5 ± 0.5	339 ± 7	782 ± 22	38. ± 0.4	-102 ± 7	-451 ± 21
	A4H8	64.9 ± 0.7	583 ± 11	1669 ± 34	64.3 ± 0.1	96.6 ± 0.8	357 ± 12	840 ± 37	31.8 ± 0.8	-226 ± 12	-829 ± 35
	T5H6	60. ± 0.4	519 ± 6	1479 ± 19	64.4 ± 0.1	94.2 ± 0.5	324 ± 8	740 ± 24	34.3 ± 0.6	-195 ± 9	-739 ± 26
$caC_{4.7}$	A8H2	64.5 ± 0.6	546 ± 9	1552 ± 27	66. ± 0.1	98.1 ± 0.6	368 ± 9	870 ± 29	33.6 ± 0.6	-178 ± 9	-682 ± 27
	A8H8	60.8 ± 0.5	505 ± 8	1433 ± 25	65.8 ± 0.1	92.3 ± 0.5	294 ± 8	650 ± 24	31.5 ± 0.5	-211 ± 8	-783 ± 23
	T9H6	62.1 ± 0.4	550 ± 7	1575 ± 21	64.1 ± 0.1	95.2 ± 0.4	347 ± 7	813 ± 20	33.1 ± 0.5	-203 ± 7	-761 ± 21
	C10H6	56.8 ± 0.6	454 ± 10	1280 ± 29	66.1 ± 0.2	92.9 ± 0.6	312 ± 9	708 ± 26	36.1 ± 0.6	-142 ± 9	-573 ± 28
	G1H8	64.6 ± 7.4	470 ± 128	1306 ± 389	75 ± 14.1	86.8 ± 4.6	122 ± 79	115 ± 241	22.2 ± 4.7	-347 ± 80	-1191 ± 244
	C2H6	74.7 ± 10.4	769 ± 158	2238 ± 475	62 ± 0.5	99.7 ± 5.6	413 ± 84	1011 ± 253	25. ± 5.5	-356 ± 83	-1227 ± 250
	A4H2	73.1 ± 1.	712 ± 16	2060 ± 49	63 ± 0.1	101.6 ± 0.6	432 ± 9	1064 ± 28	28.5 ± 0.6	-281 ± 9	-996 ± 28
	A4H8	71.9 ± 1.2	716 ± 19	2078 ± 58	62 ± 0.1	101.7 ± 1.2	444 ± 18	1102 ± 55	29.8 ± 1.1	-273 ± 18	-975 ± 53
	T5H6	64.3 ± 1.1	613 ± 18	1768 ± 53	62 ± 0.1	93.9 ± 0.8	340 ± 12	792 ± 38	29.6 ± 0.8	-273 ± 12	-976 ± 37
	C6H6	67.4 ± 1.3	652 ± 20	1886 ± 60	63 ± 0.1	99.9 ± 1.6	422 ± 24	1038 ± 73	32.5 ± 1.2	-230 ± 19	-848 ± 58
$fC_{7.0}$	C6H7	48.6 ± 1.6	344 ± 26	953 ± 78	68 ± 0.8	118.8 ± 1.8	640 ± 28	1680 ± 86	70.2 ± 1.9	296 ± 30	727 ± 90
	A8H2	71.2 ± 0.6	678 ± 9	1955 ± 28	64 ± 0.1	99.6 ± 0.4	398 ± 6	962 ± 18	28.4 ± 0.4	-280 ± 6	-994 ± 18
	A8H8	65.3 ± 0.9	604 ± 14	1737 ± 42	63 ± 0.1	98.2 ± 0.7	383 ± 11	920 ± 34	32.9 ± 0.6	-221 ± 10	-817 ± 31
	T9H6	64.6 ± 1.2	611 ± 18	1761 ± 55	63 ± 0.1	97.1 ± 1.	383 ± 16	920 ± 47	32.5 ± 1.	-228 ± 15	-841 ± 45
	C10H6	66.4 ± 1.2	636 ± 19	1835 ± 57	63 ± 0.2	97.5 ± 1.	384 ± 16	924 ± 49	31.1 ± 1.1	-252 ± 17	-911 ± 50
	C12H6	69.7 ± 1.	679 ± 15	1964 ± 47	63 ± 0.1	98.7 ± 0.9	402 ± 14	977 ± 43	29. ± 1.	-277 ± 15	-987 ± 45
	UV/Vis	50.4 ± 1.2	338 ± 25	1056 ± 77	66 ± 1.0						
